# On the role of transcription in positioning nucleosomes

**DOI:** 10.1371/journal.pcbi.1008556

**Published:** 2021-01-08

**Authors:** Zhongling Jiang, Bin Zhang

**Affiliations:** Departments of Chemistry, Massachusetts Institute of Technology, Cambridge, Massachusetts, USA; Queen’s University, CANADA

## Abstract

Nucleosome positioning is crucial for the genome’s function. Though the role of DNA sequence in positioning nucleosomes is well understood, a detailed mechanistic understanding on the impact of transcription remains lacking. Using numerical simulations, we investigated the dependence of nucleosome density profiles on transcription level across multiple species. We found that the low nucleosome affinity of yeast, but not mouse, promoters contributes to the formation of phased nucleosomes arrays for inactive genes. For the active genes, a heterogeneous distribution of +1 nucleosomes, caused by a tug-of-war between two types of remodeling enzymes, is essential for reproducing their density profiles. In particular, while positioning enzymes are known to remodel the +1 nucleosome and align it toward the transcription start site (TSS), spacer enzymes that use a pair of nucleosomes as their substrate can shift the nucleosome array away from the TSS. Competition between these enzymes results in two types of nucleosome density profiles with well- and ill-positioned +1 nucleosome. Finally, we showed that Pol II assisted histone exchange, if occurring at a fast speed, can abolish the impact of remodeling enzymes. By elucidating the role of individual factors, our study reconciles the seemingly conflicting results on the overall impact of transcription in positioning nucleosomes across species.

## Author summary

Nucleosome positioning plays a key role in the genome’s function by regulating the accessibility of protein binding sites as well as higher-order chromatin organization. Though significant progress has been made towards studying the role of DNA sequence in positioning the nucleosomes, our understanding on the impact of transcription lags behind. Our study uses kinetic simulations to explore the role of DNA sequence specificity, transcription factor binding, enzyme remodeling, and Pol II elongation in positioning nucleosomes. It suggests that the differences in nucleosome density profiles observed at various transcription levels in yeast and mouse embryonic stem cells can be understood from a tug-of-war between two types of remodeling enzymes.

## Introduction

Nucleosomes are the fundamental packaging unit of chromatin, comprising 147 base pairs (bp) of DNA wrapped around histone proteins [1]. Their formation helps to fit eukaryotic genomes inside the nucleus but also occludes the DNA binding of protein molecules, including regulatory factors and transcriptional machinery [2, 3]. The precise position of nucleosomes along the DNA sequence, therefore, can critically impact the function of the genome by regulating its accessibility [4–8]. Recent whole-genome sequencing-based studies have indeed revealed the depletion of nucleosomes at many promoter and enhancer regions to accommodate transcription [9, 10]. In addition, nucleosome positioning may affect gene expression indirectly by regulating higher-order chromatin organization [11–14]. For example, protein molecules such as Cohesin and CTCF have been shown to facilitate chromatin folding and the formation of so-called topologically associated domains [15, 16]. These domains promote enhancer-promoter contacts, and their formation relies on the accessibility of CTCF binding sites [17, 18]. Underpinning the molecular determinants of nucleosome positioning is, therefore, of fundamental interest and can provide insight into gene regulatory mechanisms.

Since the DNA molecule undergoes substantial distortion when wrapping around histone proteins, its intrinsic, sequence-specific property can impact the stability, and correspondingly position, of the formed nucleosomes [7, 19]. Numerous studies have found that nucleosomes preferentially occupy DNA segments that are more susceptible to bending and twisting. They have led to the discovery of periodic dinucleotides (AT and TA) along the nucleosome length [9, 20, 21] and intrinsically stiff poly(dA:dT) tracts at nucleosome-depleted regions [22]. Computational models based on such sequence features have been developed to predict *in vivo* nucleosome occupancy [23–25]. Accuracy of such predictions can be hampered, however, by the presence of a variety of processes and activities in the nucleus that may overwrite intrinsic positioning signals from the DNA [26–29].

**Fig 1:**
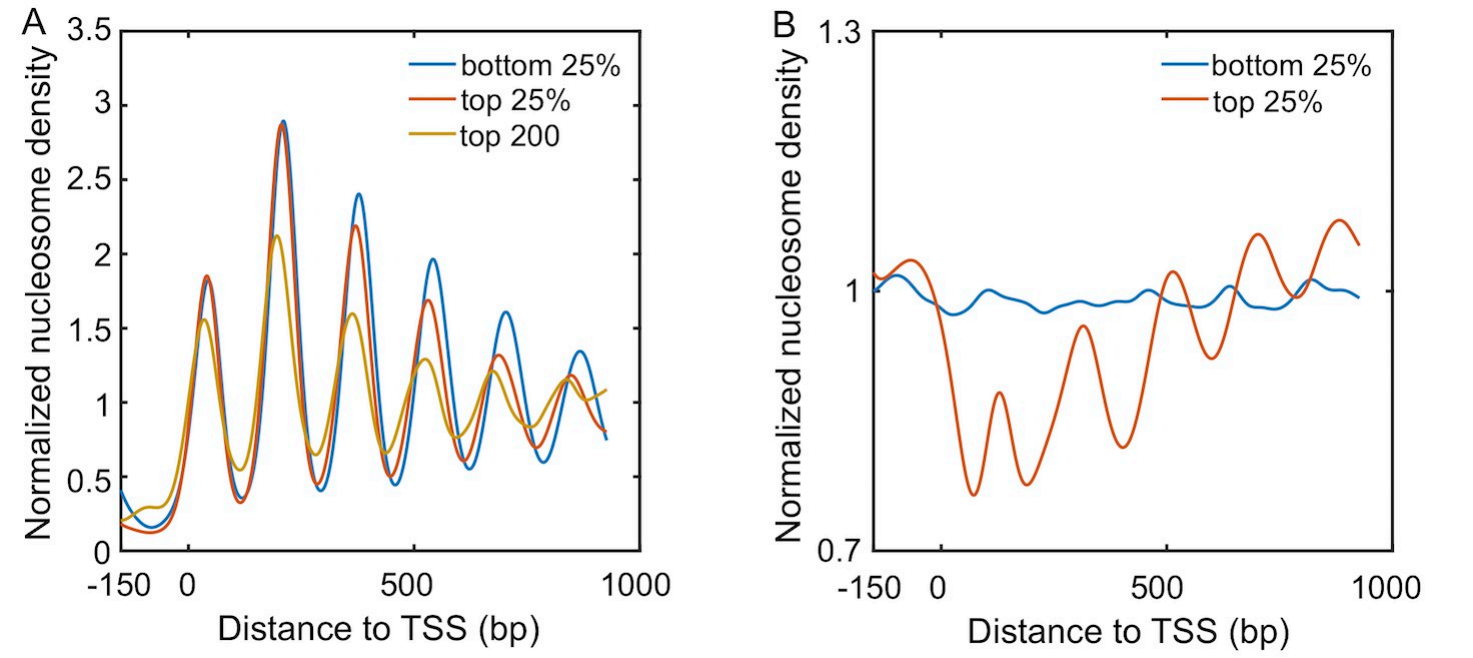
Normalized nucleosome density profiles obtained with a chemical mapping method for *S. cerevisiae* [34] (A) and mouse embryonic stem cells [31] (B) near TSS. After removing genes with more than one promoter [31, 35], 4151 and 18969 genes were considered here for *S. cerevisiae* and mouse, respectively. Genes were separated into quartiles depending on levels of transcription activities, with the bottom and top 25% corresponding the most inactive and active genes, respectively. Result for the top 200 most active genes for yeast is also shown to highlight the decrease in amplitude with increased transcriptional activity. The density profiles were smoothed with the formula Eq. S1 in the S1 Supporting information. For the raw plots, see S1 Fig.

Transcription is one of such processes that can alter the location of nucleosomes via chromatin remodeling and histone eviction [30]. Due to the consumption of ATP, the kinetics of these movements does not necessarily satisfy detailed balance, and the resulting nucleosome configurations may conflict with the thermodynamic distribution determined from the DNA sequence alone. The impact of transcription is evident from Fig 1 and S2 Fig, where average nucleosome density profiles for genes with varying levels of transcriptional activity are shown to exhibit striking differences. In particular, nucleosomes for more active genes (red) appear less ordered in yeast with less pronounced peaks and valleys when compared with inactive ones (blue). This disordering is particularly striking for the the top 200 active genes (yellow). However, the opposite trend is observed for mouse embryonic stem cells (ESC), for which clear patterns emerge from a featureless profile as transcription level elevates. We note that the qualitative trends seen in mouse ESC are conserved across multi-cellular organisms [31–33].

**Fig 2:**
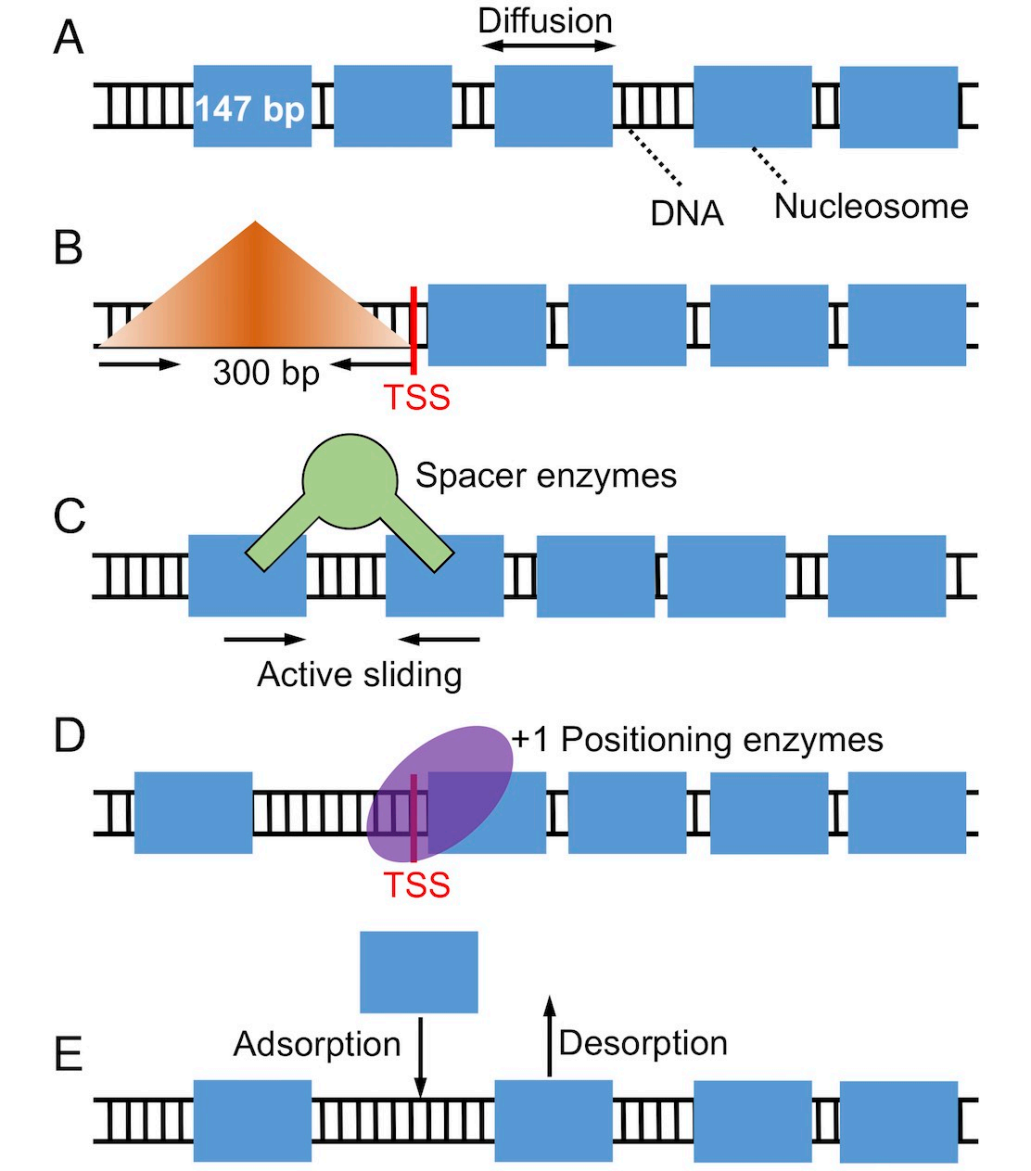
Illustration of the kinetic model used for studying nucleosome positioning that includes thermal diffusion. (A), a barrier in the promoter region that penalizes nucleosome binding (B), spacer enzyme remodeling (C), +1 nucleosome positioning, (D) and histone exchange (E). The DNA is drawn as a black ladder, and histone proteins are represented as blue rectangles. Remodeling enzymes are colored in green and use a pair of nucleosomes as substrate.

In this paper, we carried out theoretical analysis and numerical simulations of a kinetic model that explicitly considers the impact of several key factors known to impact nucleosome positioning, including the DNA sequence, chromatin remodeling enzymes, and histone exchange (Fig 2). We found that a tug-of-war between two types of remodeling enzymes explains the observed difference between nucleosome density profiles at varying transcription levels and across species. In particular, remodeling enzymes that regulate and reduce inter-nucleosome spacing tend to drive the nucleosome array away from the transcription start site (TSS). On the other hand, positioning enzymes help to align nucleosomes towards the TSS. Competition between these enzymes results in two types of density profiles with well- and ill-positioned +1 nucleosome. Mixing the two profiles at different populations can give rise to results that qualitatively reproduce yeast or mouse ESC data. We further demonstrated that fast kinetics of histone eviction/adsorption, if induced by RNA polymerase (Pol) II elongation, could reduce or abolish the impact of remodeling enzymes. Our study, therefore, provides insight into the role of transcription in positioning nucleosomes and reconciles the seemingly conflicting trend across species.

## Results

### DNA sequence contributes to the barrier for forming phased nucleosome array

A striking difference between yeast and mouse ESC is their distinct nucleosome density profiles for genes with minimal transcription activity (blue lines in Fig 1). While for yeast, these genes exhibit oscillatory patterns with well-positioned nucleosomes, the corresponding curve for mouse ESC does not show significant features. Given their low level of transcription, the impact of remodelers and transcription factors are potentially minimum. We wondered whether contributions from DNA sequences could explain nucleosome distributions in these genes.

**Fig 3:**
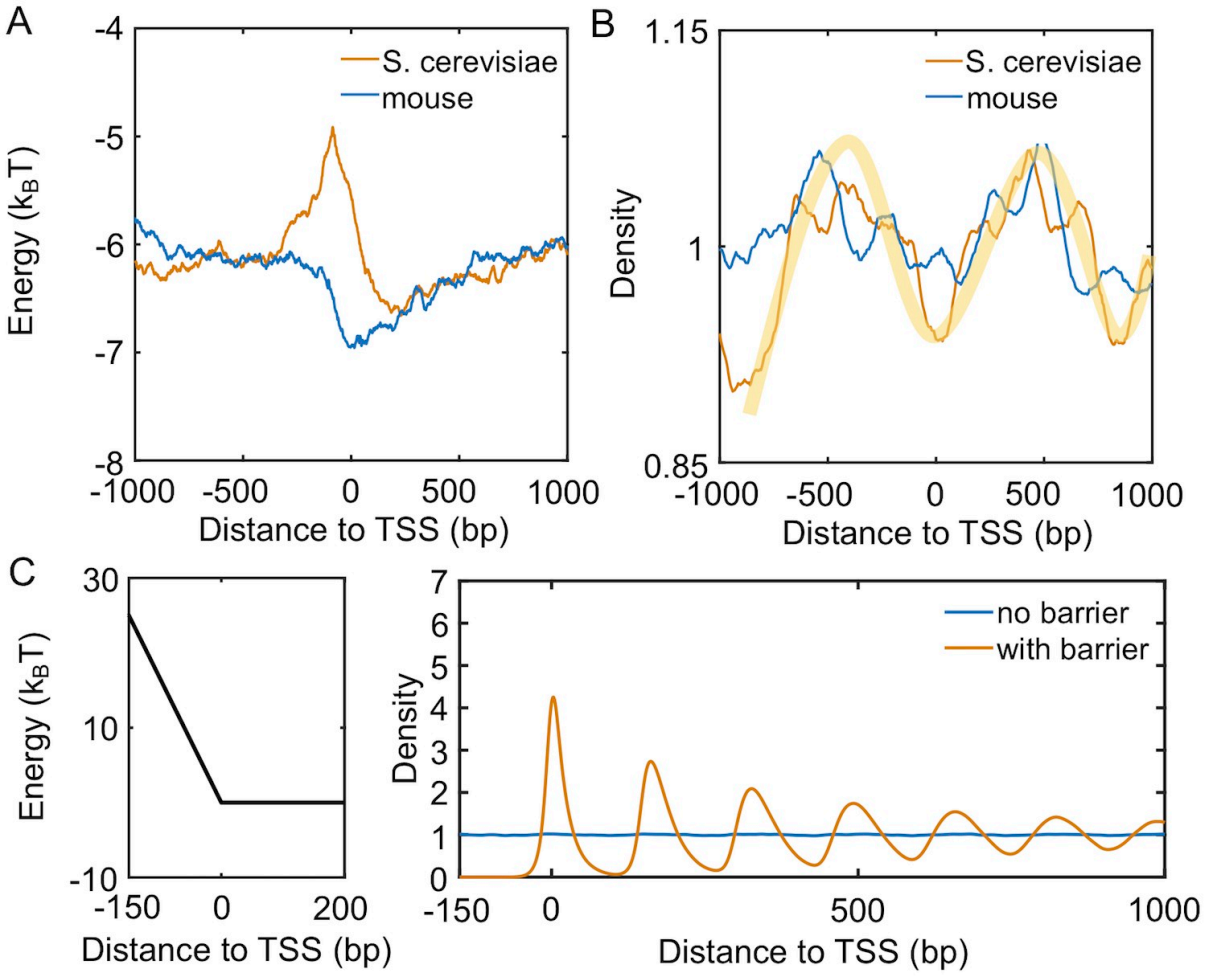
Intrinsic differences in the nucleosome affinity of promoter sequences contribute to the formation of phased nucleosome arrays in yeast, but not mouse, inactive genes. (A) Average nucleosome affinity computed for the 1000 genes with lowest transcription level for yeast (yellow) and mouse (blue). (B) Nucleosome density profiles for the corresponding sequence-specific affinity shown in part A. The shaded yellow curve is shown as a guide for the eye. (C) Nucleosome density profiles for two kinetics models that incorporate a barrier in the promoter region or not. An illustration of the promoter potential is shown on the left.

We extracted the sequences surrounding TSS for 1000 genes with the lowest transcription level from yeast and mouse genome. Using a model introduced by van Noort and coworkers that quantifies nucleosome occupancy based on a periodic function of dinucleotides [24], we determined the nucleosome affinity profile for each DNA segment. As shown in Fig 3A and S3 Fig, the average affinity for yeast genes quantified in terms of binding energy peaks at promoters located on the left of TSS. Promoters of *S. cerevisiae* are, therefore, inherently nucleosome repelling. Mouse genes, on the other hand, exhibit the opposite trend, with the same region being most favorable for nucleosome formation. The difference in promoters’ nucleosome affinity is particularly interesting in light of the statistical positioning model [10, 36], which argues that the presence of a repulsive potential could create nucleosome-free regions and align downstream nucleosomes.

To more directly evaluate the impact of DNA sequences, we carried out simulations for each inactive gene to determine their average density profiles using the predicted sequence-specific nucleosome affinity. Details for these simulations are provided in the *Materials and Methods*. As shown in Fig 3B, it is evident that for yeast but not mouse, there is a depletion of nucleosomes on the left side of TSS. This depletion gives rise to weak peaks at +1 and +2 nucleosomes. Notably, the height of the peaks do not differ significantly from the results for mouse genes and is much lower than the ones seen in Fig 1A.

The less prominent features seen in simulated density profiles are consistent with results from nucleosomes reconstituted in vitro using only genomic DNA and histones [37]. They can be attributed to the relatively small fluctuation in predicted nucleosome affinity. Additional transcription factors and remodeling enzymes could take advantage of the weakened affinity to occupy promoters [38], even for inactive genes, further driving the depletion of nucleosomes and effectively raising the barrier height. Without over complicating the model, we incorporated the effect of these proteins with a repulsive potential. As shown in Fig 3C, it similarly increases from TSS to the center of the promoter region (-150 bp) as in yeast affinity profile but with a larger slope. For simplicity, we further removed DNA-sequence specific interactions and applied uniform nucleosome binding energy across the lattice for all downstream analysis (see Table in S1 Table). Simulations carried out with the promoter potential and a uniform binding energy resulted in a density profile with clear oscillatory patterns and amplitudes comparable to those seen in experiments. If the promoter potential was removed, the simulations produced a uniform density profile as expected. Therefore, the intrinsic property of yeast promoter sequences and the binding of additional protein molecules, or a lack thereof, help recreate the nucleosome density profiles of inactive genes.

### Spacer enzymes induce nucleosome condensation and ill-positioned +1 nucleosome

We next focus on the impact of transcription on nucleosome occupancy in mouse. Fig 1B suggests that as the transcription level increases, nucleosomes become more aligned, as evidenced by the emergence of peaks and valleys. This change could arise from the establishment of nucleosome-free regions at gene promoters to accommodate the arrival of the transcription machinery. Two additional features of the density profile cannot be readily explained by the statistical positioning model, however. First, compared to the curves for yeast (Fig 1A) and from simulations (Fig 3C), the +1 nucleosome in mouse shows a much lower occupancy. Second, the spacing between nucleosomes decreases with the increase of transcriptional activity (S2(B) Fig). Here nucleosome spacing is measured as the distance between two neighboring peaks. Its decrease has also been confirmed in a recent single-cell study that directly measured the distance between nucleosomes from the same DNA molecule [39]. In the following, we explore mechanisms in addition to statistical positioning that can explain these two features.

We note that the decrease of inter-nucleosome spacing upon transcription is indeed a conserved phenomenon and can be readily seen from the yeast profiles as well. In addition, nanopore sequencing of long DNA segments that contain multiple nucleosomes has confirmed the same trend in Drosophila [32]. A possible explanation for the spacing change is the recruitment of spacer enzymes to actively transcribed genes. These enzymes use a pair of nucleosomes as their substrate and act as rulers to adjust the length of the linker DNA [27, 37, 40, 41]. Numerical simulations have confirmed their impact on inter-nucleosome distances via examining the so-called radial distribution profile [42–44]. The impact of these spacer enzymes on nucleosome density profiles near TSS remains unclear, however.

**Fig 4:**
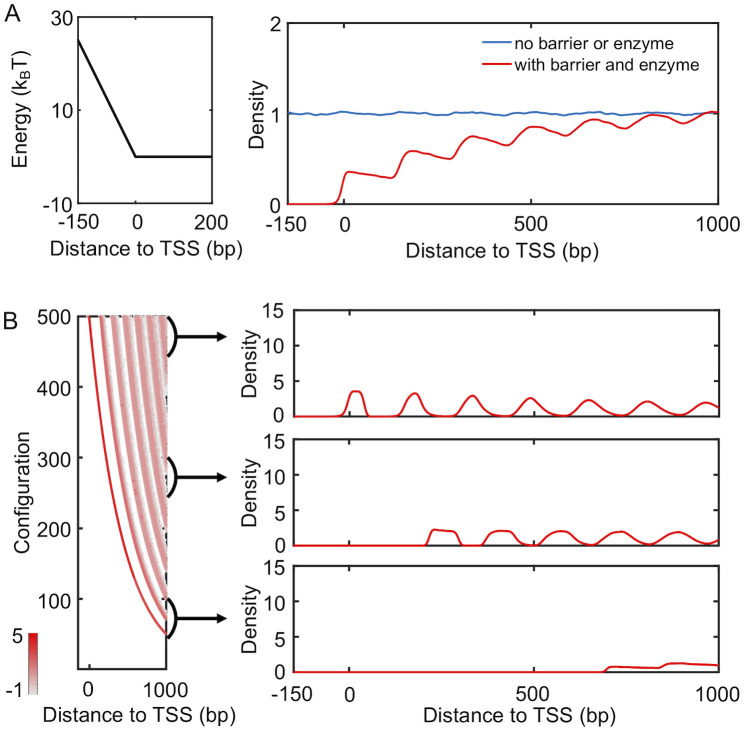
Spacer enzymes drive nucleosome condensation and the formation of ill-positioned +1 nucleosome. (A) Simulated nucleosome density profile using a model that includes a promoter potential (left) and spacer enzymes reproduce results for mouse active genes. The no barrier result is identical to that shown in Fig 3C. (B) Nucleosome configurations exhibit a wide distribution of positions for the +1 nucleosome. A scatter plot for the density distribution of all simulated configurations ordered by the position of the first nucleosome is shown on the left. Each row was computed via averaging over 1000 independent configurations. The right panel presents three example one-dimensional profiles in which the +1 nucleosome gradually shifts away from the TSS.

We carried out simulations to study the distribution of nucleosomes with the presence of spacer enzymes and a promoter potential. As detailed in the *Materials and Methods* section, these enzymes bind with a pair of neighboring nucleosomes and move them closer by one bp at every step. The rate of such remodeling steps is independent of the underlying energy landscape, and the enzymes break the detailed balance. Using a theory developed by us [44], we mapped the non-equilibrium model with enzymes onto an equivalent and renormalized equilibrium system with effective, attractive interactions between nucleosomes. To determine the distribution of nucleosomes for this effective equilibrium system, we used artificial dynamics that significantly reduces the computational time needed for statistical convergence. As shown in S4 Fig, the average distance between neighboring nucleosomes indeed decreases upon the introduction of enzymes. To our surprise, however, the density profile resembles that for active genes from mouse (see Fig 4A). In contrast to the typical decreasing trend seen in Fig 3C, the height of individual peaks gradually increases as they move away from TSS. We note that the presence of the barrier potential is crucial to reveal the effect of spacer enzymes. Simulations that include spacer enzymes but without the barrier potential produced a density profile resembling the one for silent genes, i.e., the no barrier or enzyme case (S5 Fig). For simulations of genes with intermediate expression level and weakened barrier potential and enzyme remodeling rate, see S6 Fig.

Examining the simulated nucleosome arrays revealed a wide range of configurations with both well- and ill-positioned +1 nucleosome. We first ordered the nucleosome arrays along the *y*-axis based on the position of the first nucleosome and computed the corresponding local nucleosome density profiles. The results are shown in the left panel of Fig 4B, with representative, traditional one-dimensional profiles presented on the right. The top configurations exhibit a well-defined +1 nucleosome, and the corresponding density profile resembles that of a statistical positioning model shown in Fig 3C. For many of the configurations near the bottom of the plot, the +1 nucleosome shifts away from the TSS, giving rise to a wide nucleosome free region. A mixture of these configurations with varying +1 nucleosome positions results in the final profile shown in Fig 4A.

The inclusion of spacer enzymes can, therefore, impact both inter-nucleosome distances and the position of the +1 nucleosome. Without these enzymes, nucleosomes will occupy all accessible DNA regions while staying as far apart from each other as possible to maximize entropy. This tendency for an equal partition of the DNA is the essence of the statistical positioning model. It will ensure the confinement of the +1 nucleosome in a narrow region between the TSS and the +2 nucleosome. On the other hand, spacer enzymes introduce effective attraction between nucleosomes and cause them to aggregate rather than staying farther apart [44]. The entire array of nucleosomes now behaves as a single entity, and individual nucleosomes are no longer uniformly distributed across the genome. The free, collective movement of the entire nucleosome array with respect to the TSS, again driven by entropy, will result in ill-positioned +1 nucleosome.

### A mixture of profiles with well- and ill-positioned +1 nucleosome reproduces yeast results

The presence of a promoter potential and spacer enzymes leads to the formation of two types of nucleosome density profiles with well- and ill-positioned +1 nucleosome. A mixture of the two types qualitatively reproduces the experimental results for active mouse genes. We next investigated whether the same mixture but with different levels of population can explain yeast nucleosome density profiles.

The more pronounced patterns seen in yeast profiles suggest that configurations with well-positioned +1 nucleosome should dominate. We note that many positioning enzyme are known to align nucleosomes toward the TSS [37]. To mimic the impact of these molecules, we introduced an additional positioning potential between the region of 0 and 30 bp from TSS. This approximate treatment avoids making explicit assumptions regarding the molecular mechanisms of remodeling enzymes that remain largely unknown.

**Fig 5:**
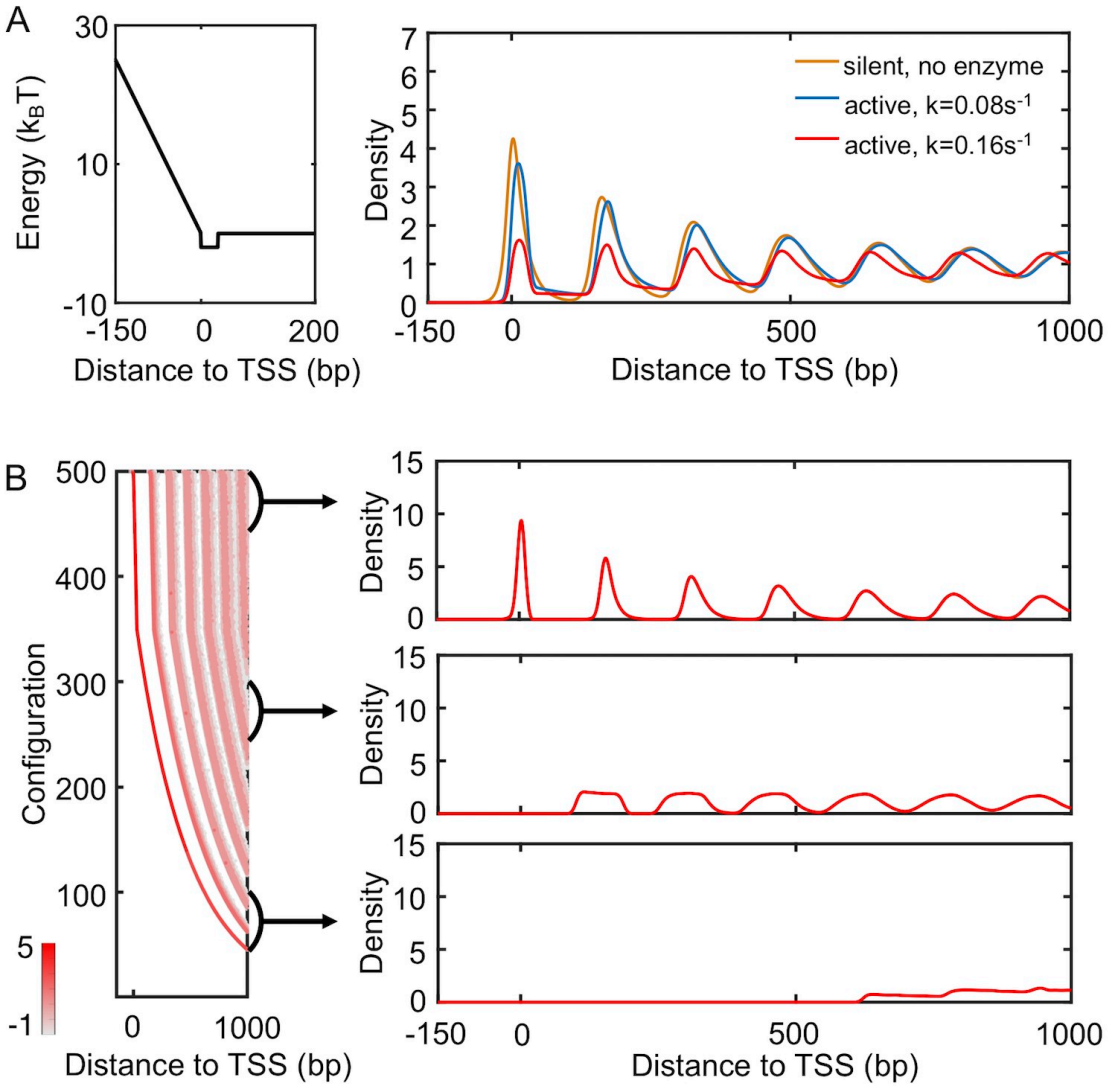
A mixture of configurations with well- and ill-positioned +1 nucleosome reproduce yeast density profiles. (A) Nucleosome density profiles determined with the presence of a positioning potential of -2*k*_*B*_*T* introduced to the region following the promoter (left). Comparison between the two density profiles with varying rates for spacer enzymes confirms that more active genes with higher enzyme rates exhibit lower peaks. The yellow curve from Fig 3C that corresponds to the profile of yeast silent genes is shown for reference. (B) Scatter plot for the density distribution of all simulated configurations ordered by the position of the +1 nucleosome (left), with example one-dimensional profiles shown on the right. Each row was computed via averaging over 1000 independent configurations.

As shown in Fig 5, the new potential succeeds in attracting nucleosomes to the TSS. The resulting density profile (red) now resembles those from yeast and the height of the peaks decreases as they move away from the TSS. We note that as transcription activity decreases, enzymes will be recruited less to the genes, and their effective remodeling rate will be smaller. Slower spacer enzymes with a rate of *k* = 0.08*s*^−1^ compete less effectively with the positioning enzymes, and the relative population of configurations with ill-positioned +1 nucleosome decreases. Correspondingly, the nucleosome profile exhibits higher peaks (blue) than that for more active genes with a faster enzyme rate, consistent with the dependence on transcription activity seen in experimental results (Fig 1A).

Introducing the positioning potential also breaks the symmetry across the TSS (S7 Fig). The nucleosome density profile on its left hand side is less prominent as the one on the right and shown in Fig 5A. This asymmetry is qualitatively consistent with experimental observations. It is worth mentioning that the yeast results shown in Fig 1A deviate from the simulated profiles in that the second peak is higher than the first. However, this same trend was not observed in recent single-molecule studies that simultaneously measure the position of multiple nucleosomes from the same DNA [45, 46]. Additional experiments are needed to evaluate the robustness and mechanism of this particular feature further.

### Fast histone exchange abolishes the impact of spacer enzymes

For highly transcribed genes, in addition to remodeling enzymes, Pol II could impact the positioning of nucleosomes as well. As it elongates along the DNA, Pol II could cause partial or complete loss of histone proteins [28, 47–49]. In the following, we investigate the impact of Pol II induced histone eviction on nucleosome density profiles.

Specifically, we carried out stochastic simulations that explicitly model nucleosome diffusion, enzyme remodeling, and histone eviction and absorption. We assumed that the eviction and absorption rates depend only on inter-nucleosome and nucleosome-DNA interactions and are independent of remodeling enzymes (see Materials and methods). The ratio of the two was tuned to ensure a density of approximately 0.88. The basal rate constant *r*_const_ = 0.1*s*^−1^ was estimated from the transcription rate 1 min^−1^ for the most active genes [50] with the assumption of full eviction for all nucleosomes.

**Fig 6:**
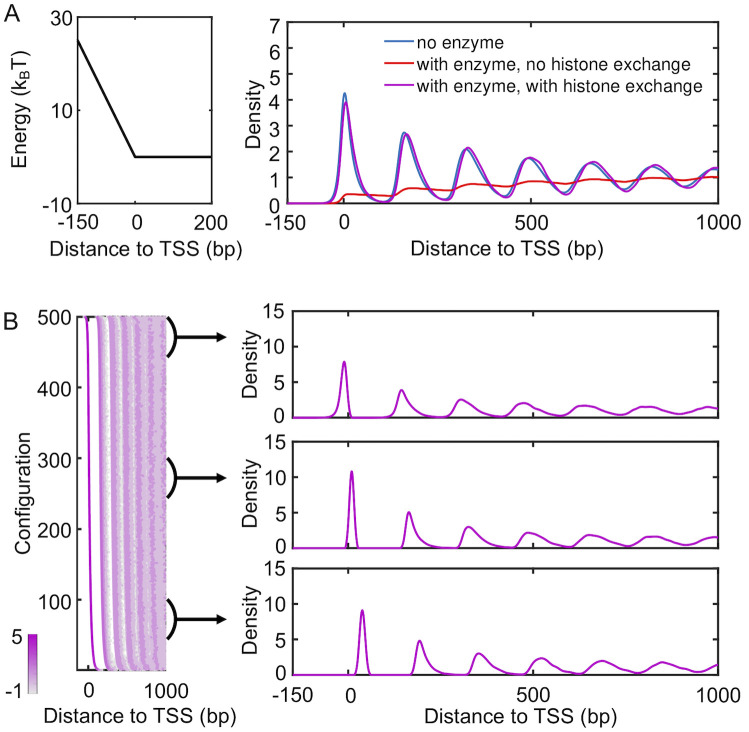
Impact of histone exchange kinetics on nucleosome density profiles. (A) Nucleosome density profiles determined from kinetic models that only includes a promoter potential (blue), that includes both a promoter potential and spacer enzymes (red), and that includes a promoter potential, spacer enzymes and histone exchange (purple). The red curve is identical to the one shown in Fig 4A. An illustration of the promoter potential is shown on the left. (B) Scatter plot for the density distribution of all simulated configurations ordered by the position of the +1 nucleosome (left), with example one-dimensional profiles shown on the right. Each row was computed via averaging over 1000 independent configurations.

The resulting nucleosome density profile is shown in Fig 6 (purple). It differs significantly from the one obtained from a kinetic model that only included remodeling enzymes and a promoter potential (red), which was also shown in Fig 4A. The impact of remodeling enzymes in these simulations is significantly reduced. In particular, a pronounced peak emerges near TSS, and the density profile now traces well the result from a model with only the promoter potential (blue). The decrease of inter-nucleosome spacing cannot be observed in the radial distribution profile either (S8 Fig). We found that the impact of histone eviction on the density profile depends on its rate and gradually diminishes as the rate slows down (S9 Fig). It is worth noting that a significantly smaller rate (10^−8^*s*^−1^) is needed to reveal the impact of remodeling enzymes on nucleosome spacing. Since a decrease of inter-nucleosome spacing is readily seen in experimental nucleosome density profiles, we anticipate that complete eviction of histone octamers to be rare [28, 51–53].

The competition between Pol II and spacer enzymes on positioning nucleosomes can be understood as following. The diffusive dynamics driven by thermal motions defines an equilibrium distribution of nucleosomes along the DNA. This distribution depends both on inter-nucleosome and nucleosome-DNA interactions. Spacer enzymes modify this distribution by introducing an effective attractive potential between nucleosomes. The two-dimensional dynamics of histone exchange can give rise to, yet, another steady-state distribution. Unless slowed down substantially, histone exchange can lead to faster relaxation kinetics when compared with nucleosome movements restricted to one dimension. It will essentially overwrite any impact caused by spacer enzymes or diffusion on nucleosome distribution. If the rates for histone eviction and adsorption satisfy detailed balance with regard to the normal potential for nucleosome-DNA and inter-nucleosome interactions, the steady-state distribution determined from histone exchange kinetics should be consistent to the equilibrium distribution obtained from pure diffusion. This consistency explains the agreement between purple and blue lines seen in Fig 6A. On the other hand, if the two rates were modified to account for the effective interaction potential induced by spacer enzymes, the steady-state distribution will reproduce the one dictated by spacer enzymes. Such kinetics, though less meaningful biologically, can prove beneficial for reducing the computational cost of stochastic simulations (see Materials and methods for more discussions).

## Conclusions and discussion

In this paper, we investigated the impact of transcription on nucleosome positioning. By partitioning genes based on their transcriptional activity, we determined the corresponding nucleosome density profiles for both yeast and mouse. A striking difference for inactive genes was observed between the two species. Similar featureless profiles as that from mouse have been observed for inactive genes from Drosophila [32] and human [33] as well. Analyzing the nucleosome binding affinity of DNA sequences suggests that while yeast promoters are nucleosome repelling, the opposite holds true for mouse promoters. This difference could contribute to the formation of phased nucleosome arrays in yeast, but not mouse ESC, via the statistical positioning mechanism. The nucleosome attracting promoters appear to be a rule of multi-cellular organisms rather than an exception in mouse, as shown in prior studies [23, 54, 55]. They might function to suppress the expression of certain genes crucial for cell differentiation [55].

We further carried out stochastic simulations to study the variation of nucleosome density profiles as the transcriptional activity elevates. Focusing on qualitative trends rather than quantitative agreements allowed us to extract a minimalist model of nucleosome positioning. We discovered that a tug-of-war between two types of enzymes is the key to rationalize the observed trends. In particular, enzymes that use a pair of nucleosomes as substrate can induce nucleosome condensation and tend to shift the nucleosome array away from TSS, giving rise to density profiles with ill-positioned +1 nucleosome. Positioning enzymes, on the other hand, can counteract this effect and align the +1 nucleosome back to TSS. A combination of density profiles with well- and ill-positioned +1 nucleosome can qualitatively reproduce *in vivo* results from both yeast and mouse ESC.

Possible candidates for spacer enzymes include Chd1 and ISW1 and INO80 may function as positioning enzymes. Density profiles determined from nucleosomes reconstituted *in vitro* with and without these enzymes indeed appear to consistent with our model predictions [37]. However, validating the impact of these enzymes *in vivo* can be complicated by the presence of additional enzymes with redundant roles. In addition, some of the enzymes have been reported to play significant roles both in positioning the +1 nucleosome and regulating nucleosome spacing.

We note that many details on nucleosome positioning remain unknown. When designing the kinetic model, we opted for simplicity and interpretability over quantitative accuracy. In particular, we did not explicitly consider DNA sequence effect for results shown in Figs 4 and 5. To examine the robustness of our conclusions on remodeling enzymes, we performed additional simulations that explicitly incorporated sequence specific nucleosome binding energy profiles. As shown in S10 Fig, we were able to qualitatively reproduce the same trends using models that incorporate nucleosome binding affinity of *S. cerevisiae* and mouse ESC genes (see Table in S2 Table). Spacer enzymes were able to create ill-positioned +1 nucleosomes even with the presence of DNA sequence specific binding, while positioning enyzmes remain successful at aligning the +1 nucleosomes towards TSS.

**Fig 7:**
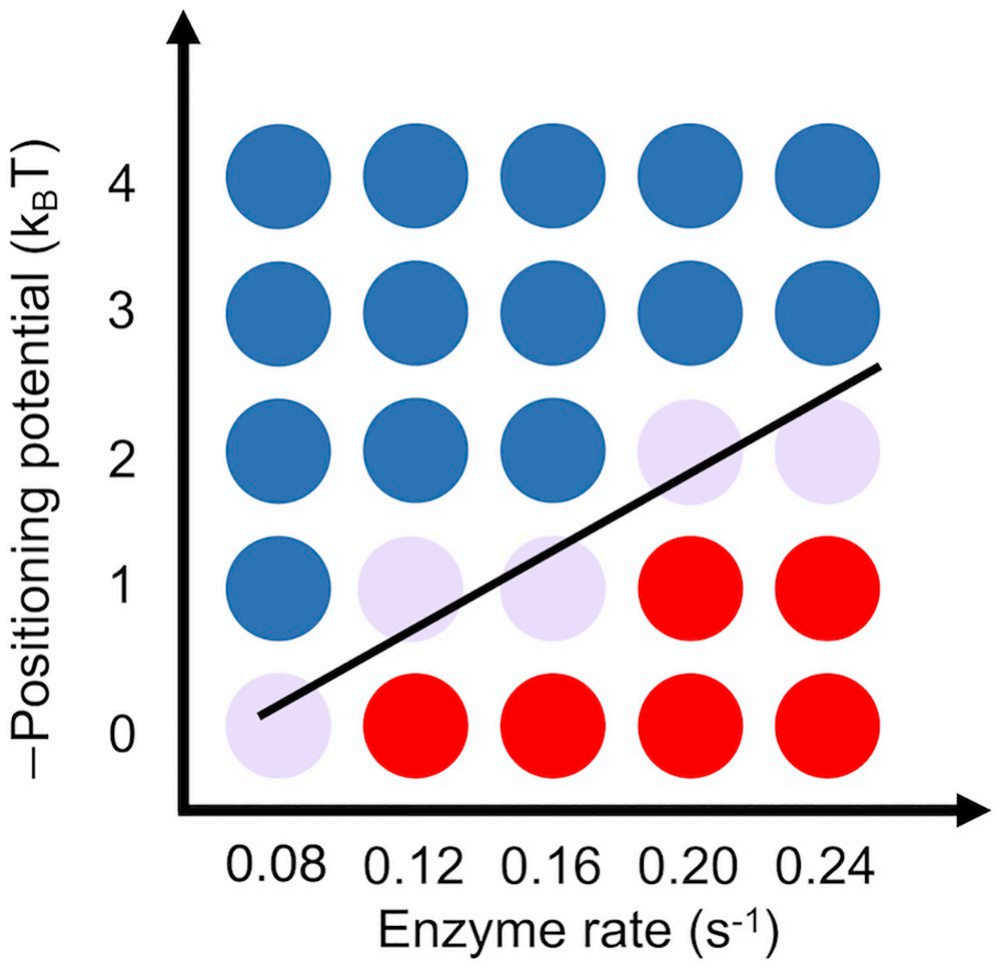
Phase diagram of nucleosome density profile with respect to the strength of the positioning potential and the rate of spacer enzymes. Blue and red dots correspond to yeast and mouse-like patterns, respectively. Each dot represents an independent stochastic simulation using the parameters specified in the two axes. We defined mouse-like nucleosome density profiles as the ones with a gradual increase of peak height, i.e., the 2nd peak is higher than the 1st one. Yeast-like nucleosome density profiles as the ones with a gradual decrease of peak height, i.e., the 2nd peak is lower than the 1st one. The black line connecting the purple dots represents the phase transition line.

To evaluate the robustness of our conclusions on model parameters, we computed a phase diagram with respect to the rate of spacer enzymes and the strength of the positioning potential. As shown in Fig 7, the competition between two opposing forces that favor yeast and mouse-like density profiles is evident and can be seen across a wide range of parameters.

A direct consequence of the competition between the two types of remodeling enzymes is the heterogeneity in the position of +1 nucleosomes, as is evident in Figs 4 and 5. Also, compared to yeast, the +1 nucleosomes from mouse ESC should exhibit a larger variance. Similarly, active genes in yeast are predicted to possess less well-defined +1 nucleosome positions than the inactive ones. We note that the position of individual +1 nucleosomes cannot be determined from the bulk data analyzed in Fig 1. To validate these predictions, we analyzed single-molecule data produced by Zhao [39] and Au group [45]. As shown in S11 and S12 Figs, consistent with model predictions, there is indeed a significant variance in the +1 nucleosome position for individual genes from both species. The relative trend between mouse and yeast genes and between yeast active and inactive genes is consistent with model predictions. The single-molecule results, therefore, provide strong support for the mechanism uncovered from our simulations.

It is worth emphasizing that in addition to the competition between remodeling enzymes, other factors could contribute to the experimental results. For example, we focused our analysis on a single gene, but the results shown in Fig 1 are from averaging over many genes. In particular, individual genes could differ in the range of the promoter potential shown in Fig 4A due to the binding of a diverse set of transcription factors [38]. This variation in promoter potential could result in further heterogeneity of the +1 nucleosome and the lowering of the corresponding peak in the density profile. Accounting for such gene-specific features, though will not impact the conclusions here based on common features shared by all genes, will be crucial for predicting nucleosome positioning in silico.

## Materials and methods

### Kinetic model of nucleosome positioning

We consider a one-dimensional lattice model to study the positioning of nucleosomes along the DNA sequence (Fig 2). Each lattice site *s* represents a single bp and is assigned with a nucleosome binding energy *V*_*s*_. In most cases, *V*_*s*_ was set to a constant value, such that the nucleosome binding energy of a 147 bp long DNA segment is *V*_*i*_ = −42*k*_*B*_*T* [56], to focus on the impact of remodeling enzymes. When the DNA sequence effect was explicitly considered, we determined *V*_*s*_ using the periodic function of dinucleotides introduced by van Noort and coworkers [24]. The length of the lattice is 14700 bp, and the periodic boundary condition was enforced to eliminate any end effect. Nucleosome density was set as 0.88, a typical value found near the gene coding regions in yeast [53].

To account for the excluded volume effect, a pair potential was introduced between neighboring nucleosomes *i* and *i* + 1 as

$$\begin{array}{c} u\left(\Delta x_{i}\right)=\{\begin{array}{cc} \text{infinity} & \Delta x<147\\ 0 & \text{otherwise}, \end{array} \end{array}$$ (1)

where Δ*x*_*i*_ = *x*_*i*+1_ − *x*_*i*_. *x*_*i*_ corresponds to the dyad position of the *i*-th nucleosome, and by definition, each nucleosome occupies a region of 147 bp in length [1].

Nucleosomes can move along the DNA via diffusive motion with a rate of

$$\begin{array}{c} d=\frac{D}{{\left(\Delta l\right)}^{2}}e^{-\beta \Delta U/2}, \end{array}$$ (2)

where *D* = 1bp^2^/s is the diffusion coefficient and Δ*l* = 1bp is the step size [57]. Δ*U* denotes the change of the total energy before and after nucleosome movement. The rate expression was designed such that detailed balance is satisfied [56].

In addition to thermal motions, positions of nucleosomes can be altered by transcription related activities as well [58]. In the following, we consider the impact of three major factors related to transcription.

First, transcription factors, preinitiation complex, and Pol II are known to compete with histone proteins to bind gene promoters [38, 59]. We incorporated these proteins’ effect as an energetic barrier centered at 150 bp upstream of TSS to penalize nucleosome formation. Similar treatment has been used by Padinhateeri and coworkers to create a nucleosome-free region near TSS [60]. As illustrated in Fig 2B, the barrier is symmetric with respect to the center. Its triangular shape allows nucleosomes to occupy the promoter region with a finite probability. The mathematical expression for this promoter potential is provided in the S1 Supporting information.

Second, active transcription can recruit remodeling enzymes to alter the position of nucleosomes at the expanse of ATP. While several types of remodeling enzymes have been discovered, here we focus on ISW1-like enzymes that modulate inter-nucleosome spacing and INO80-like enzymes that adjust the position of the +1 nucleosome [27, 37, 40, 61–64]. Following Möbius *et al.* [43], we assumed that spacer enzymes bind to neighboring nucleosomes that are within 332 bp at a rate of 0.16 *s*^−1^ [62, 65], and randomly move one of them toward the other by one bp (Fig 2C). For the positioning enzymes, we modeled their effect with a positioning potential located near TSS.

Finally, transcription of the gene body by Pol II could displace nucleosomes completely off the DNA [28, 47]. To account for such disrupt events, we explicitly modeled absorption and desorption of histone proteins with rate expressions

$$\begin{array}{c} r_{\text{on}}=r_{\text{const}}e^{-\beta \Delta V/2},\;r_{\text{off}}=r_{\text{const}}e^{\beta \Delta V/2}. \end{array}$$ (3)

*r*_const_ is the rate constant, and Δ*V* = Δ*U* + *μ* includes both the change in the system’s total energy Δ*U* and a chemical potential *μ*. The impact of Pol II was incorporated into the rate constant and the chemical potential, whose value was tuned to ensure an average system density of 0.88 (see Table in S1 Table).

### Details of stochastic simulations

We carried out stochastic simulations using the Gillespie algorithm [66] to determine steady-state nucleosome density profiles.

#### Simulations without Pol II facilitated histone exchange

In several of the kinetic models explored in the *Results* Section, the effect of histone eviction from Pol II was not explicitly considered. Without remodeling enzymes, these models describe systems with equilibrium statistics since the diffusive dynamics follows detailed balance (Eq 2). When remodeling enzymes are present, as shown in our previous study [44], the kinetic model can be rigorously mapped onto an effective equilibrium system with renormalized temperature and potential, detailed expressions for which are provided in the SI. For such *one-dimensional* equilibrium or quasi-equilibrium systems, there is a well defined, unique distribution for each model that depends only on inter-nucleosome potentials and DNA sequence. These distributions are independent of the kinetic schemes used in stochastic simulations as long as they satisfy detailed balance. Therefore, for their determination, we simulated only “artificial” absorption and desorption kinetics with rates defined in Eq 3 and *r*_const_ = 12*s*^−1^. Renormalized potentials were used to determine the change in the system’s total energy Δ*U* if remodeling enzymes were introduced in the kinetic model. The two-dimensional dynamics for histone exchange helps to alleviate the topological constraint and jamming dynamics experienced if the system is restricted to one dimension with all nucleosomes bound to the DNA. It can significantly reduce the computational time needed for convergence. In the large number limit, the statistics of the grand canonical ensemble with histone exchange should be equivalent to that of a system restricted to one dimension with fixed nucleosome number. In S13 Fig, we showed that, for the system size considered here, the fluctuation in nucleosome number is small and has minimal impact on the resulting density profile.

We carried out 200 independent simulations for kinetic models lacking DNA sequence specificity. To investigate the impact of DNA sequences, we also separately carried out 1000 simulations for both yeast and mouse. Each one of these simulations incorporates a nucleosome binding affinity profile predicted from the sequence of an inactive gene. All simulations lasted for 5000 seconds and were initialized with over 80 nucleosomes randomly distributed over the lattice. 2500 configurations were recorded every two seconds in each simulation to determine the density profiles.

#### Simulations with Pol II facilitated histone eviction

Pol II and remodeling enzymes can evict and assemble nucleosomes during transcription [47, 49]. This two-dimensional kinetics for histone exchange defines a steady-state distribution consistent with the reaction rates defined in Eq 3. The one-dimensional spacer enzymes, by themselves, can give rise to another steady-state distribution that depends on enzyme kinetics. If the rate expressions for histone exchange were modified to account for the effective interaction induced by enzymes, the two steady-state distributions are consistent with each other. This consistency inspired our use of artificial exchange kinetics to accelerate computer simulations mentioned above. Biologically, however, histone exchange rates most likely do not depend on spacer enzymes, and the two distributions will be in conflict.

To rigorously account for the impact of both kinetics, we performed stochastic simulations that explicitly include diffusion, enzyme remodeling, and histone eviction and absorption as well. A total of 2500 independent 5 × 10^5^-second-long simulations were performed. Only 200 configurations in the last 400 seconds of each simulation were collected with an equal time interval to compute the density profiles. These simulations were again initialized with randomly placed nucleosomes over the lattice.

### Data processing

Genome-wide mappings of nucleosome positions obtained with a chemical mapping method are available for *S. cerevisiae* and mouse embryonic stem cells in the NCBI database with accession number GSE36063 and GSE82127. Compared to the micrococcal nuclease digestion, followed by high-throughput sequencing (MNase-seq) [29], the chemical mapping approach is affected less by sequence preference or nucleosome unwrapping and can provide base pair resolution of nucleosome center positions [31, 34]. We point out, however, that the qualitative trend shown in Fig 1A has been observed for data obtained with MNase-seq as well [30].

To determine the transcription level of individual genes, we downloaded RNA-seq data using accession number GSE52086 for yeast [67] and GSE82127 for mouse [31]. Genes with more than one promoter [31, 35] were removed from analysis. DNA sequences surrounding TSS were extracted from the Eukaryotic Promoter Database [68, 69] based on the ID provided in the RNA-seq data.

## Supporting information

S1 Supporting information Supporting information for “On the role of transcription in positioning nucleosomes”. (PDF)

S1 Table Values of the chemical potential used to ensure an average density of 0.88. *V*_*i*_ = −42k_B_T is the equilibrium nucleosome binding free energy [56], and *V*_*i*_ + *μ* provides an estimation of the effective nucleosome affinity in each model.
(PDF)

S2 TableValues of the chemical potential used to ensure an average density of 0.88 or 0.78 on simulations with DNA sequence specific nucleosome binding.
$\bar{V_{i}}$ is the mean nucleosome binding free energy of each gene calculated with the model introduced by van Noort and coworkers [24], and $\bar{V_{i}}+\mu$ provides an estimation of the effective nucleosome affinity in each model.(PDF)

S1 Fig The raw nucleosome density profiles shown in Fig 1 of the main text before smoothing. See text *Nucleosome density profile smoothing* in S1 Supporting information for details on the smoothing function.
(PDF)

S2 Fig Normalized nucleosome density profiles for *S. cerevisiae* and mouse near TSS. (A) *S. cerevisiae* [34]. (B) mouse [31]. After removing genes with more than one promoter [31, 35], 4151 and 18969 genes were considered here for *S. cerevisiae* and mouse, respectively. These genes were separated into quartiles depending on their transcription activities, with the bottom and top 25% corresponding to the most inactive and active genes, respectively. For *S. cerevisiae*, using the first five peaks for each curve, we estimated the inter-nucleosome spacing as 164.75 bp for bottom 25%, 165.25 bp for bottom 25%-50%, 163.75 bp for top 25%-50%, 161.5 bp for top 25%, and 159.75 bp for top 200. For mouse, the spacing was similarly estimated as 189.75 bp for top 25%-50% and 188.5 bp for top 25%.
(PDF)

S3 Fig Average nucleosome affinity computed for *S. cerevisiae* and mouse genes with different expression levels. (A) *S. cerevisiae*. (B) mouse. The same procedure as in Fig 3A of the main text was used in computing these profiles.
(PDF)

S4 Fig Comparison between radial distribution profiles for kinetic models with or without spacer enzymes and average nucleosome spacing as a function of the remodeling rate of spacer enzymes. (A) Comparison between radial distribution profiles for kinetic models with (red) or without (blue) spacer enzymes. The plots were computed from the same data used in Fig 4A of the main text. It is evident that, upon the introduction of spacer enzymes, inter-nucleosome distances decrease. See *text: Definition of the density profile and radial distribution function* in S1 Supporting information for more details. (B) Average nucleosome spacing as a function of the remodeling rate of spacer enzymes. The spacing was determined using the average distance between neighboring nucleosomes. The nucleosome pair flanking the TSS was excluded from distance estimation.
(PDF)

S5 Fig Nucleosome density profile obtained from simulations that include spacer enzymes but not the barrier potential. Nucleosome density profile obtained from simulations that include spacer enzymes but not the barrier potential (blue), which was obtained by averaging 500 independent 5000-second-long simulations to achieve a better ensemble average under the strong clustering effect of spacer enzymes. For reference, the profile determined with the presence of both the barrier potential and spacer enzymes (red), i.e., the red curve in Fig 4A of the main text, is also shown.
(PDF)

S6 Fig Simulated nucleosome density profile for mouse genes with intermediate expression level (purple). For reference, the corresponding profile of silent (blue) and active (red) genes, i.e., the two curves in Fig 4A of the main text, are also shown. We note that unlike yeast, mouse inactive genes differ from the active genes in two ways. First, mouse inactive genes do not have a well defined nucleosome free region nor barrier potential. Second, mouse inactive genes lack the remodeling enzymes that are known to be associated with transcription. This is the key reason that we used “no barrier or enzyme” in Fig 4A. Yeast inactive genes, on the other hand, do have a well defined nucleosome free region and barrier potential as evidenced in Fig 1A. Because of the presence of differences in both barrier potential and remodeling enzymes between mouse active and inactive genes, studying “middle” genes in mouse requires tuning two parameters rather than one single parameter as in yeast. In the simulations used for intermediate genes, we decreased the height of the barrier potential from 25 to 2*k*_B_*T* and reduced the rate of spacer enzymes from 0.16 to 0.12 *s*^−1^. Other simulation setups were kept the same as for active genes.
(PDF)

S7 Fig The nucleosome density profile across both sides of the TSS exhibits asymmetric patterns. The same data for the blue curve in Fig 5A of the main text was used here to determine the density profile.
(PDF)

S8 Fig Fast histone change kinetics abolishes the impact of remodeling enzymes on inter-nucleosome spacing. To measure the inter-nucleosome spacing, we determined the radial distribution profiles from the same data used to compute the nucleosome density profiles shown in Fig 6A of the main text. The result from a kinetic model with spacer enzymes and histone exchange (purple) is almost identical to that from a model with no enzymes (blue) and does not exhibit any decrease in inter-nucleosome spacing.
(PDF)

S9 Fig Dependence of nucleosome density profile and inter-nucleosome spacing on the rate of histone exchange kinetics that was varied from 10^−2^ *s*^−1^, to 10^−6^ *s*^−1^ and to 10^−8^ *s*^−1^. (A) 10^−2^ *s*^−1^. (B) 10^−6^ *s*^−1^. (C) 10^−8^ *s*^−1^. The radial distribution profiles for the kinetic models without enzyme and with enzyme but no histone exchange are identical to those shown in S8 Fig.
(PDF)

S10 Fig Nucleosome density profiles determined using DNA sequence-specific binding energy support the robustness of conclusions presented in the main text. (A) The corresponding plot to Fig 3C of the main text. The blue line was computed without the barrier potential and is identical to the mouse result shown in Fig 3B of the main text. We used the nucleosome binding profiles of mouse genes in the corresponding simulations. The red line was obtained from simulations with the barrier potential and the nucleosome binding profiles of *S. cerevisiae* genes. (B,C) The corresponding plots to Fig 4A of the main text. Simulations performed for part B used the same set up as those in the main text, except with the addition of nucleosome binding profiles computed using mouse genes. Simulations performed for part C uses a shifted promoter potential and a nucleosome density of 0.78. Both of these two changes are supported by the experimental data shown in Fig 1 of the main text. (D) The corresponding plot to Fig 5A of the main text. Simulations were performed using the same set up as those in the main text, except with the addition of nucleosome binding profiles computed using yeast genes. See text *Simulations with DNA sequence specific nucleosome binding* in S1 Supporting information for simulation details.
(PDF)

S11 Fig Single-cell data supports the heterogeneous distribution of the +1 nucleosome position in mouse ESC. The single-cell nucleosome mapping data for mouse ESC was obtained from the NCBI database with accession number GSE96688 [39]. The +1 nucleosome was defined as the first nucleosome in the downstream 1000 bp region of TSS. Only mapped nucleosomal fragments with a length of 140-180 bp were retained, and the centers of fragments were considered as the nucleosome dyad position. We further discarded segments for which the first nucleosome is more than 1000 bp away from the TSS. This treatment left out 121 genes (18,969 in total) for which no nucleosomes were found in all samples within 1000 bp of TSS. (A) Genes exhibit large variance in the position of the +1 nucleosomes. Each row represents an individual gene, and they were sorted based on the mean distance between +1 nucleosome and TSS. The solid line correspond to the mean position and the light blue errorbars represent standard deviations. (B) Probability distribution of the variance shown in part A. (C) Probability distribution of distance between +1 nucleosome position of a given gene *x*[*n*] and the smallest position of that gene found across all cells *x*[1]. We note that a potential caveat of single cell experiments is that they might suffer from sparse data coverage. Failing to detect some nucleosomes could misguide data analysis, causing the assignment of +2 or +3 as +1 nucleosome and an exaggeration of the variance seen in part A. Though it would be difficult to rule out artifacts from missing data, we can gauge their impact by considering extreme cases. In particular, we consider the case that all nucleosome arrays in different cells have identical +1 nucleosome positions, and the variance arises purely from missing data and misassignment. In this case, the probability distribution of *x*[*n*] − *x*[1] should exhibits clear peaks separated by nucleosome repeat length. As shown in part C, no significant peaks can be seen in the distribution. Therefore, the +1 nucleosome position variance should not arise purely as a result of data artifact, but reflects the true heterogeneity across cells.
(PDF)

S12 Fig Single molecule data supports the heterogeneous distribution of the +1 nucleosome position in *S. cerevisiae*. Data produced from the Au group [45] were used for the analysis. The +1 nucleosome was defined as the first nucleosome found in the [-50, 1000] bp region around TSS. We included 50 bp upstream of the TSS since the +1 nucleosomes peak in yeast is closer to the TSS than mouse ESC, as is evident in Fig 1 of the main text. (A) Variance of +1 nucleosome position across different genes. Each row represents an individual gene, and they were sorted based on the mean distance between +1 nucleosome and TSS. The solid line correspond to the mean position and the light blue errorbars represent standard deviations. (B,C) Probability distribution of the +1 nucleosome position for various group of genes.
(PDF)

S13 Fig Accuracy of the simulation algorithm with “artificial” kinetics for histone absorption/desorption. (A) Probability distribution for the number of nucleosomes bound to the DNA computed from the simulation data used for creating Fig 4A (red) of the main text. Over 90% of the simulated configurations falls into the ±10 interval of the peak nucleosome number 86. We note that the length of free DNA region is only 98 nucleosomes due to the presence of the barrier potential in the promoter region. The peak of the distribution with 86 nucleosomes, therefore, provides a density of approximately 0.88. (B) Comparison between nucleosome density profiles obtained from artificial kinetics (red) and from rigorous stochastic simulations of diffusion and enzyme remodeling (blue). In these additional simulations, no explicit histone exchange was considered, and the 86 nucleosomes remain bound to the one-dimensional lattice at all time. We performed 500 simulations that lasted for 7.5 × 10^6^ seconds and were initialized from randomly distributed nucleosomes. 1000 configurations were extracted from the last 5 × 10^5^ s of each trajectory at every 500 seconds to compute the density profile. The good agreement between the two density profiles provide strong support for the accuracy of the algorithm with artificial kinetics used in the main text.
(PDF)
