# Supplementary material for: On the role of transcription in positioning nucleosomes
Source: PLoS Comput Biol. 2021 Jan 8;17(1):e1008556. doi: 10.1371/journal.pcbi.1008556 (PMC7819601; doi:10.1371/journal.pcbi.1008556)
Supplement: S1 Supporting information — (PDF) [file pcbi.1008556.s001.pdf]

Supporting Information for “On the role of transcription in positioning nucleosomes”

Zhongling Jiang and Bin Zhang*

Departments of Chemistry, Massachusetts Institute of Technology, Cambridge, MA, USA

E-mail: binz@mit.edu

Nucleosome density profile smoothing

The nucleosome density profiles ($\mathrm{n}(i)$) shown in Fig 1 of the main text were directly computed from experimental data.[1,2] Following Voong et al., we further smoothed the curves using the following formula

$$\mathrm{n}(i) = \sum_{j=-73}^{j=73} \mathrm{S}(i+j)e^{-(j/20)^2/2}, \tag{S1}$$

where $\mathrm{S}(i+j)$ is the original nucleosome density data before smoothing. This smoothing does not impact the qualitative trends seen in Fig 1, but helps for robustly identifying the peaks and estimating inter-nucleosome spacing.

All the simulated nucleosome density data were similarly smoothed with the default Gaussian-smooth function in Matlab over data points within a 31-bp window.

Barrier potential for nucleosome depletion

A linear potential was introduced upstream of the transcription start site (TSS) for creating nucleosome free regions. It was defined with the following expression.

$$V_{\mathrm{b}}(s) = \begin{cases} -h & 0\text{ bp} \le s < 30\text{ bp} \\ \frac{1}{6}(150 - |s+150|) & -300\text{ bp} \le s \le 0\text{ bp} \\ 0 & \text{otherwise.} \end{cases} \tag{S2}$$

$h = 2k_BT$ corresponds to the strength of the positioning potential for aligning nucleosomes towards the TSS.

Expressions for renormalized temperature and potential from remodeling enzymes

In Ref. 3, we showed that the non-equilibrium model that includes the kinetics of remodeling enzymes can be rigorously mapped onto an effective equilibrium with renormalized temperature

and potential defined as following

$$u^{\text{eff}}(\Delta x_i) = \begin{cases} \text{infinity} & \Delta x_i < 147\text{bp} \\ \frac{k}{2}\overline{C}[(\Delta x_i - \Delta x_{\max}) - \frac{1}{2}] & \Delta x_i \leq \Delta x_{\max} \\ 0 & \text{otherwise.} \end{cases} \tag{S3}$$

$\Delta x_i = x_{i+1} - x_i$ is the position difference between the i-th nucleosome and its upstream neighbor. k is the enzyme rate. $\overline{C}$ is the ensemble average of $C_{i,i+1}$, which equals 1 if neighboring nucleosomes are within $\Delta x_{\max} = 332$ bp and 0 otherwise. The effective temperature $T_{\text{eff}} = \frac{T}{D} \cdot (D + \frac{1}{2}kl^2\overline{C})$. Since our system density is around 0.88, the average nucleosome spacing is 165 bp, which is much smaller than 332 bp. Hence, we approximated $\overline{C}$ as one.

Definition of the density profile and radial distribution function

We used both the density profile and the radial distribution function to characterize the simulated configurations of nucleosomes. While the density profile measures the absolute position of nucleosomes along the lattice, the radial distribution function emphasizes on the inter-nucleosome distance and the relative position of nucleosomes with respect to each other. The mathematical definitions for them are provided below.

The one dimensional normalized density profile is defined as

$$\rho(r) = \left\langle \frac{\sum_i^N \delta(r_i - r)}{N/L} \right\rangle, \tag{S4}$$

where L = 100-nucleosome (i.e. 14700 bp) is the total length of simulated system. i indexes individual nucleosomes and N is the total number of nucleosomes. r_i corresponds to the central position of nucleosome i. Since we kept the overall density N/L in all simulations to be ~ 0.88, the normalization does not influence the peak height comparison among various density profiles.

The radial distribution function is defined as

$$g(r) = \left\langle \frac{\sum_{i=1}^{N} \sum_{j>i}^{N} \delta(|r_i - r_j| - r)}{N^2/L} \right\rangle. \tag{S5}$$

The variables are similarly defined as in Eq. S4.

Simulations with DNA sequence specific nucleosome binding

To examine the robustness of our conclusions on remodeling enzymes, we performed additional simulations that explicitly incorporated sequence specific nucleosome binding energy profiles. In particular, we recreated results that are equivalent to those shown in Figs 3C, 4A, and 5A of the main text. The resulting nucleosome density profiles are shown in S10 Fig.

Each curve shown in S10 Fig were obtained from averaging over 1000 independent simulations. In each one of the simulations, we incorporated a binding energy profile determined using one of the 1000 genes with the lowest transcription level from either *S. cerevisiae* or mouse. We further varied the value of the chemical potential (S2 Table) to achieve the desired nucleosome density. Simulations were initialized with 80 randomly distributed nucleosomes and lasted for 5000 seconds. The first 100 seconds were discarded as equilibration.

S10A Fig corresponds to Fig 3C of the main text. The blue line was computed without the presence of the barrier potential and is identical to the mouse result shown in Fig 3B of the main text. We used the nucleosome binding profiles of mouse genes in the corresponding simulations. The density profile is relatively flat across the whole range, as is the case for mouse inactive genes. The red line was obtained from simulations with the barrier potential and the nucleosome binding profiles of *S. cerevisiae* genes. The barrier potential succeeds in creating oscillatory patterns.

S10B Fig corresponds to Fig 4A of the main text. The upward trend of peak heights in the red curve is slightly less evident than that shown in the main text. This difference can be understood from the average nucleosome binding profile (Fig 3B of the main text). The presence of an attractive well essentially pulls the nucleosome cluster closer towards the TSS.

The difference between the two, however, can be largely resolved by further tuning model parameters. In particular, the center of the promoter potential and the nucleosome density in the model were both determined based on yeast profiles. However, the nucleosome depletion region for mouse centers near TSS, rather than 150 bp upstream of TSS as in yeast (Fig 1 of the main text). Furthermore, the average nucleosome spacing in mouse is around 188 bp, giving rise to a density of $\rho = 147/188 = 78\%$, which is smaller than 88% from yeast. If these two parameters were included into the model, the resulting nucleosome density profile (S10C Fig) is indeed more similar to the experimental result and the upward trend in peak heights is restored.

S10D Fig corresponds to Fig 5A of the main text. The simulation setup is identical in the two cases. We used the nucleosome binding profiles of yeast genes in the new simulations. The positioning enzymes again succeed at aligning the +1 nucleosome towards TSS, while a faster spacer enzyme can lower the peak heights and reduce nucleosome spacing.

Therefore, our conclusions on the role of a promoter potential, spacer enzymes, and positioning enzymes in nucleosome density profiles hold true even with the presence of DNA sequence. These new simulation results support that the variation in nucleosome density profiles for genes with different transcription levels, or the variation for genes across species with similar transcription levels, are not directly driven by the differences of the underlying DNA sequence. Instead, a tug-of-war between two types of remodeling enzymes can explain the experimental trends qualitatively.

References

(1) Brogaard, K.; Xi, L.; Wang, J.-P.; Widom, J. A map of nucleosome positions in yeast at base-pair resolution. *Nature* **2012**, *486*, 496–501.

(2) Voong, L. N.; Xi, L.; Sebeson, A. C.; Xiong, B.; Wang, J.-P.; Wang, X. Insights into nucleosome organization in mouse embryonic stem cells through chemical mapping. *Cell* **2016**, *167*, 1555–1570.

(3) Jiang, Z.; Zhang, B. Theory of active chromatin remodeling. *Phys. Rev. Lett.* **2019**, *123*, 208102.
