# Supplementary material for: On the role of transcription in positioning nucleosomes
Source: PLoS Comput Biol. 2021 Jan 8;17(1):e1008556. doi: 10.1371/journal.pcbi.1008556 (PMC7819601; doi:10.1371/journal.pcbi.1008556)
Supplement: S1 Table — Vi = −42kBT is the equilibrium nucleosome binding free energy [56], and Vi + μ provides an estimation of the effective nucleosome affinity in each model. (PDF) [file pcbi.1008556.s002.pdf]

Kinetic Model	V_i+μ (k_BT)
no barrier (Figure 3C blue)	-4.2
with barrier (Figure 3C, yellow)	-3.8
no barrier or enzyme (Figure 4A, blue)	-4.2
with barrier and enzyme (Figure 4A, red)	17.3
silent, no enzyme (Figure 5A, yellow)	-3.8
active, $k = 0.08s^{-1}$ (Figure 5A, blue)	9.5
active, $k = 0.16s^{-1}$ (Figure 5A, red)	17.33
no enzyme (Figure 6A, blue)	-3.8
with enzyme, no histone exchange (Figure 6A, red)	17.3
with enzyme, with histone exchange (Figure 6A, purple)	-3.4
with enzyme, with histone exchange (Figure S5A, purple)	-3.4
with enzyme, with histone exchange (Figure S5B, purple)	-1
with enzyme, with histone exchange (Figure S5C, purple)	-1
