# Supplementary material for: On the role of transcription in positioning nucleosomes
Source: PLoS Comput Biol. 2021 Jan 8;17(1):e1008556. doi: 10.1371/journal.pcbi.1008556 (PMC7819601; doi:10.1371/journal.pcbi.1008556)
Supplement: S2 Table — Vi‾ is the mean nucleosome binding free energy of each gene calculated with the model introduced by van Noort and coworkers [24], and Vi‾+μ provides an estimation of the effective nucleosome affinity in each model. (PDF) [file pcbi.1008556.s003.pdf]

Kinetic Model	$\bar{V}_i + \mu$ ($k_B T$)
S. cerevisiae (Figure 3B yellow)	-6
mouse (Figure 3B blue)	-6
S. cerevisiae (Figure S10A, blue)	-4
mouse (Figure S10A, red)	-4
no barrier or enzyme (Figure S10B, blue)	-6
with barrier and enzyme (Figure S10B, red)	16.7
no barrier or enzyme (Figure S10C, blue)	-6
with barrier and enzyme (Figure S10C, red)	16.7
$k = 0.08 s^{-1}$ (Figure S10D, blue)	8.2
$k = 0.16 s^{-1}$ (Figure S10D, red)	16.7
