# Supplementary figures and images for: On the role of transcription in positioning nucleosomes
Source: PLoS Comput Biol. 2021 Jan 8;17(1):e1008556. doi: 10.1371/journal.pcbi.1008556 (PMC7819601; doi:10.1371/journal.pcbi.1008556)

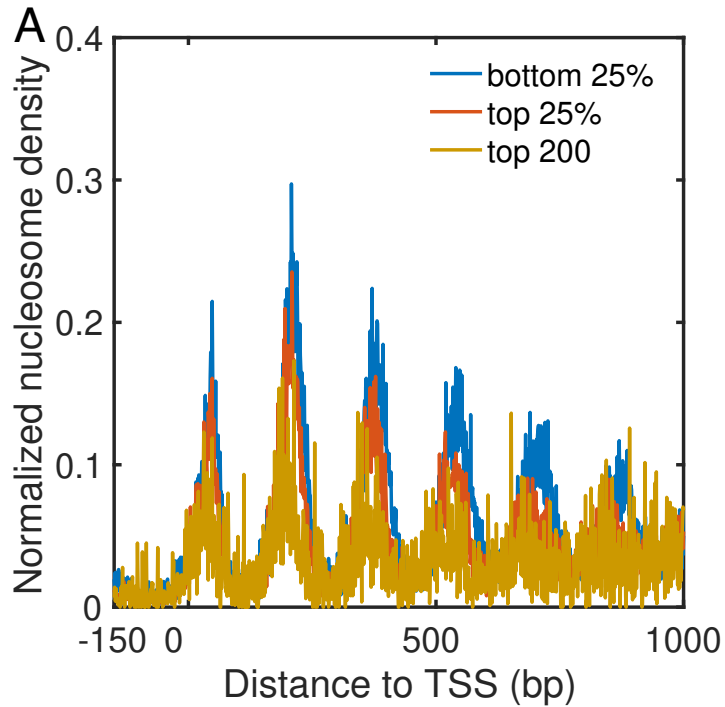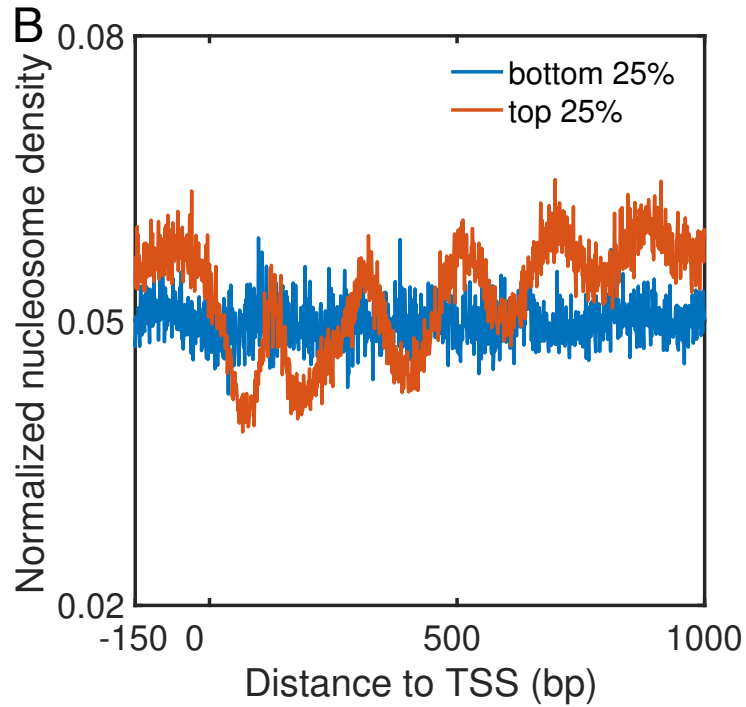

Supplement: S1 Fig — See text Nucleosome density profile smoothing in S1 Supporting information for details on the smoothing function. (PDF) [file pcbi.1008556.s004.pdf]

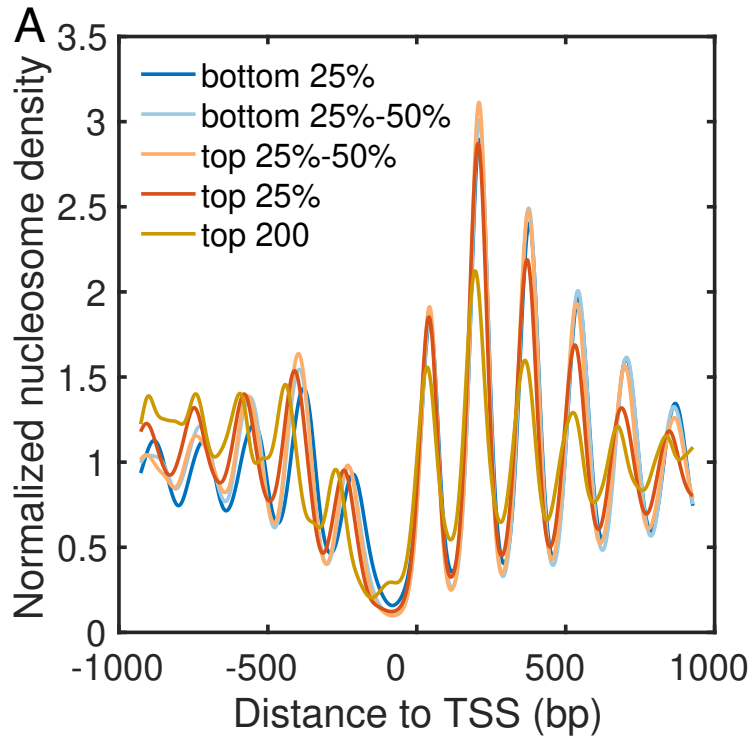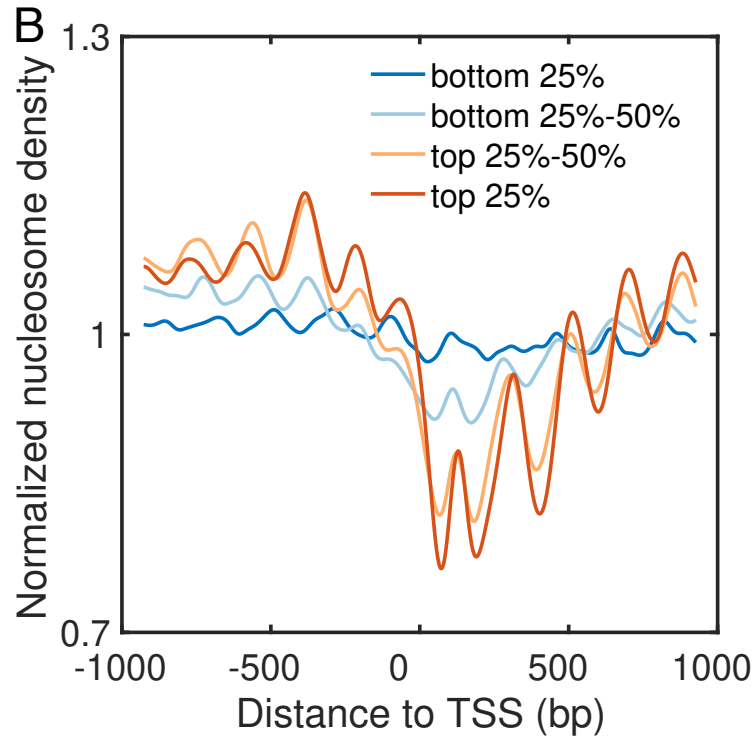

Supplement: S2 Fig — (A) S. cerevisiae [34]. (B) mouse [31]. After removing genes with more than one promoter [31, 35], 4151 and 18969 genes were considered here for S. cerevisiae and mouse, respectively. These genes were separated into quartiles depending on their transcription activities, with the bottom and top 25% corresponding to the most inactive and active genes, respectively. For S. cerevisiae, using the first five peaks for each curve, we estimated the inter-nucleosome spacing as 164.75 bp for bottom 25%, 165.25 bp for bottom 25%-50%, 163.75 bp for top 25%-50%, 161.5 bp for top 25%, and 159.75 bp for top 200. For mouse, the spacing was similarly estimated as 189.75 bp for top 25%-50% and 188.5 bp for top 25%. (PDF) [file pcbi.1008556.s005.pdf]

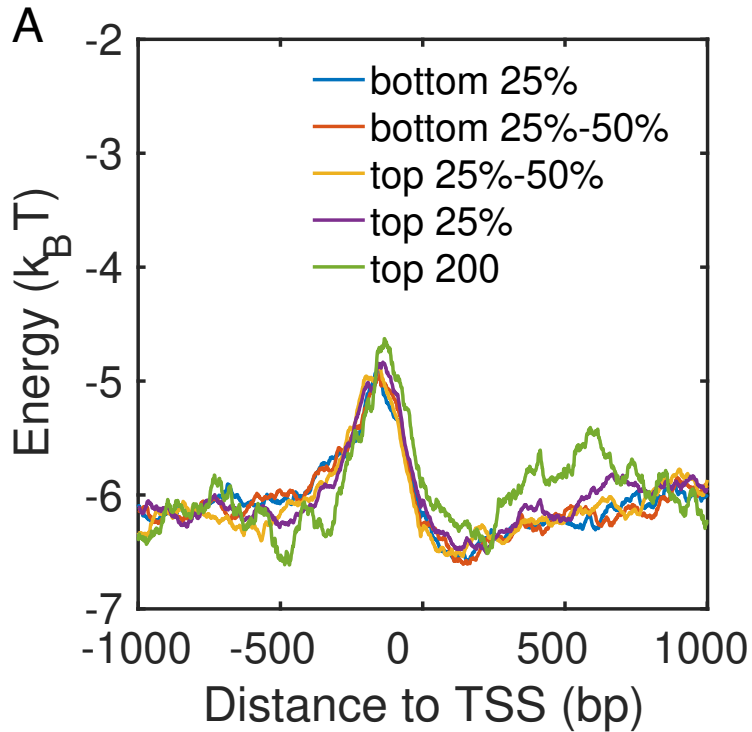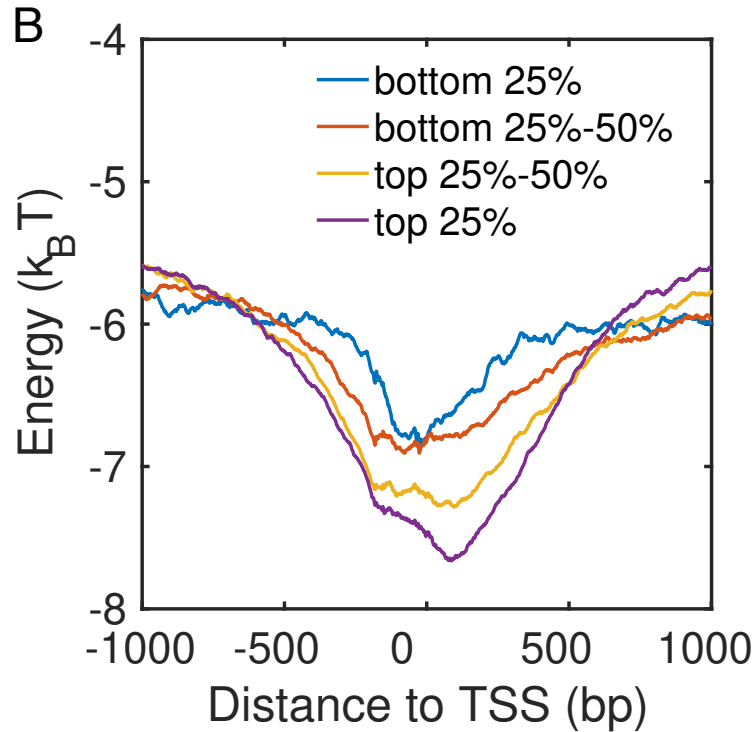

Supplement: S3 Fig — (A) S. cerevisiae. (B) mouse. The same procedure as in Fig 3A of the main text was used in computing these profiles. (PDF) [file pcbi.1008556.s006.pdf]

A

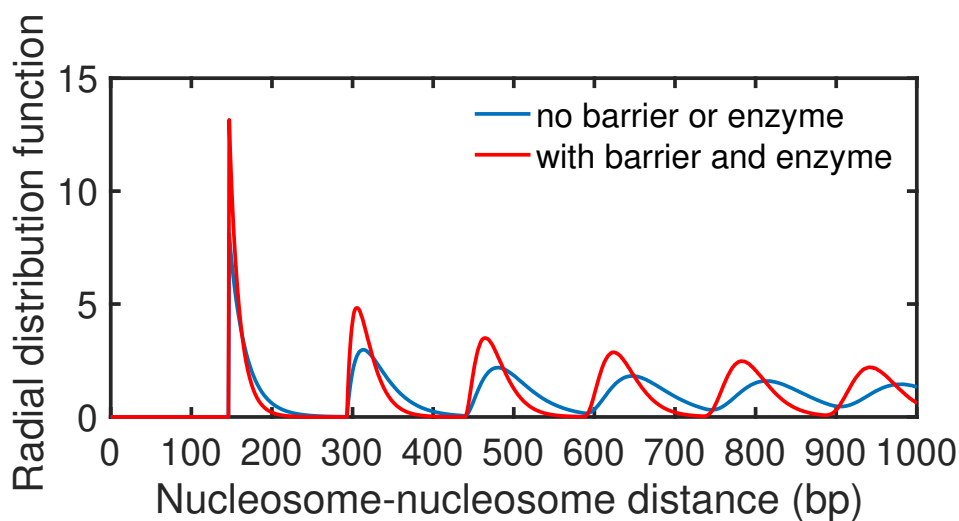

B

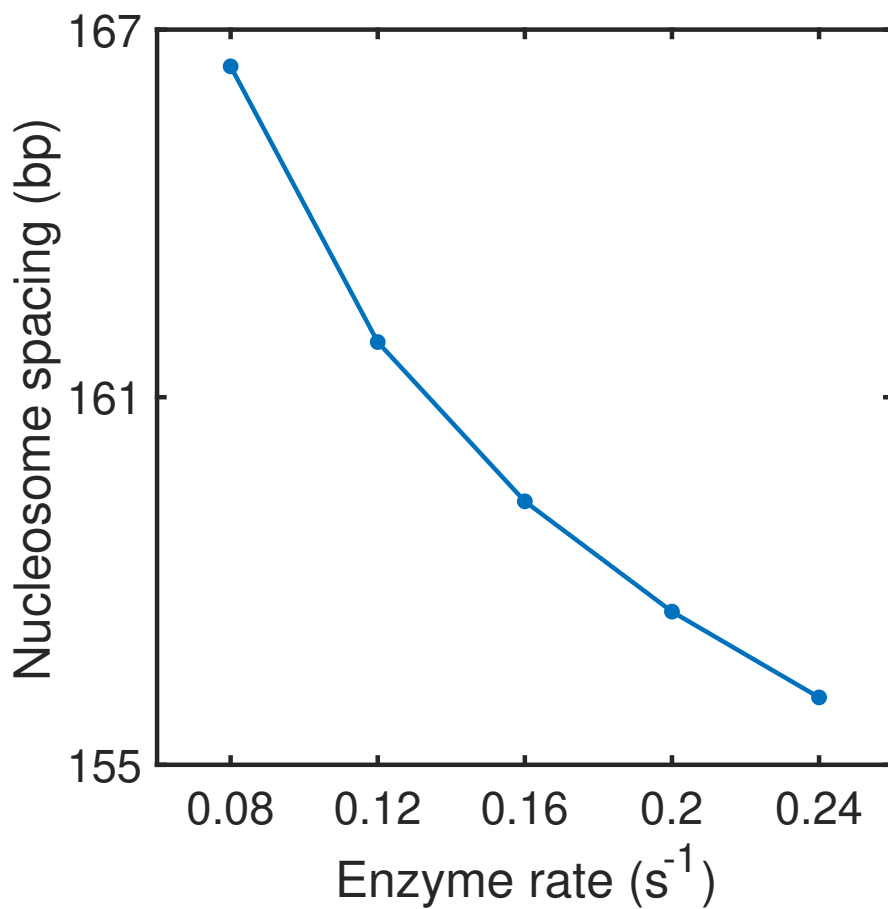

Supplement: S4 Fig — (A) Comparison between radial distribution profiles for kinetic models with (red) or without (blue) spacer enzymes. The plots were computed from the same data used in Fig 4A of the main text. It is evident that, upon the introduction of spacer enzymes, inter-nucleosome distances decrease. See text: Definition of the density profile and radial distribution function in S1 Supporting information for more details. (B) Average nucleosome spacing as a function of the remodeling rate of spacer enzymes. The spacing was determined using the average distance between neighboring nucleosomes. The nucleosome pair flanking the TSS was excluded from distance estimation. (PDF) [file pcbi.1008556.s007.pdf]

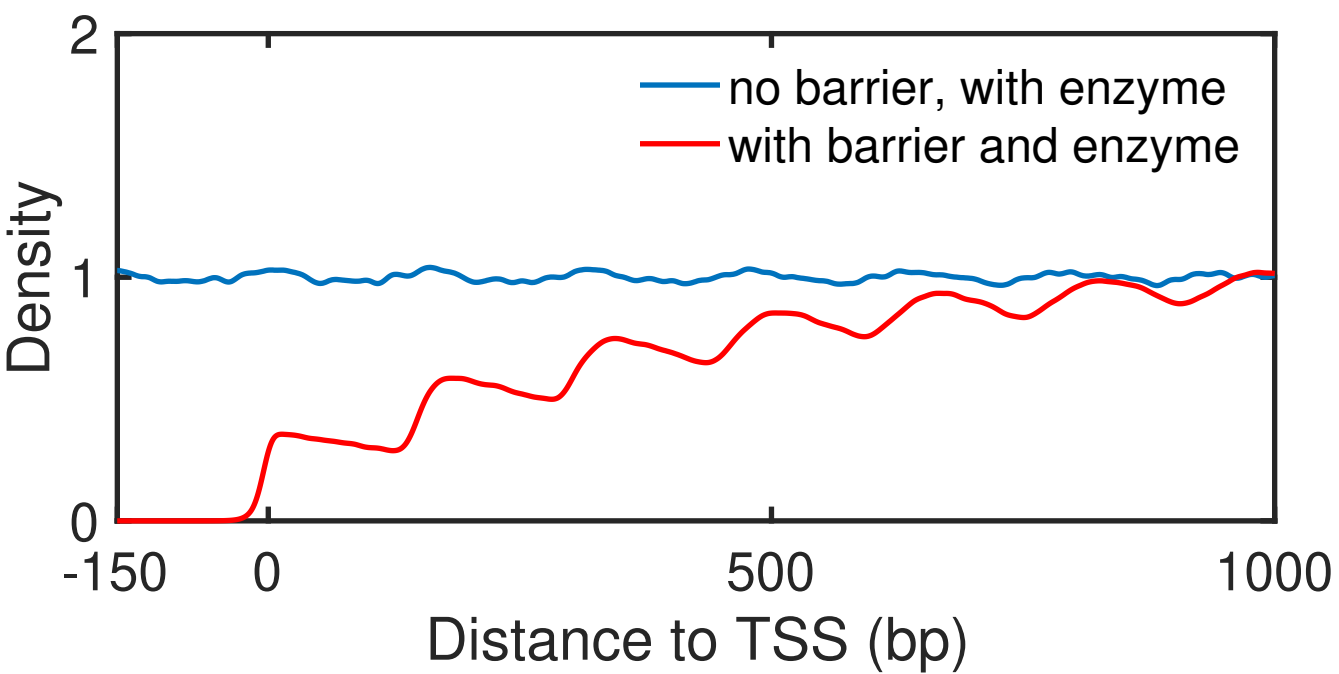

Supplement: S5 Fig — Nucleosome density profile obtained from simulations that include spacer enzymes but not the barrier potential (blue), which was obtained by averaging 500 independent 5000-second-long simulations to achieve a better ensemble average under the strong clustering effect of spacer enzymes. For reference, the profile determined with the presence of both the barrier potential and spacer enzymes (red), i.e., the red curve in Fig 4A of the main text, is also shown. (PDF) [file pcbi.1008556.s008.pdf]

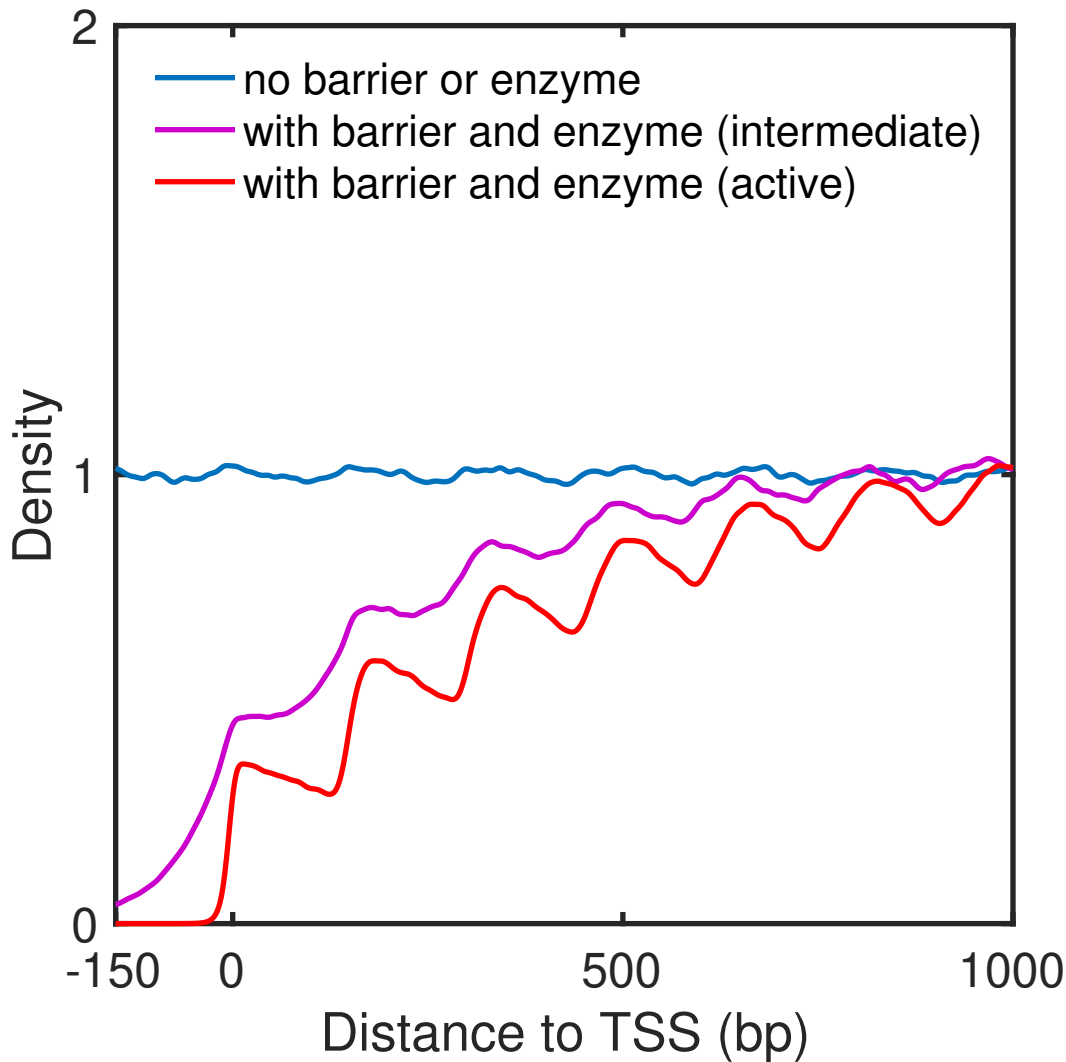

Supplement: S6 Fig — For reference, the corresponding profile of silent (blue) and active (red) genes, i.e., the two curves in Fig 4A of the main text, are also shown. We note that unlike yeast, mouse inactive genes differ from the active genes in two ways. First, mouse inactive genes do not have a well defined nucleosome free region nor barrier potential. Second, mouse inactive genes lack the remodeling enzymes that are known to be associated with transcription. This is the key reason that we used “no barrier or enzyme” in Fig 4A. Yeast inactive genes, on the other hand, do have a well defined nucleosome free region and barrier potential as evidenced in Fig 1A. Because of the presence of differences in both barrier potential and remodeling enzymes between mouse active and inactive genes, studying “middle” genes in mouse requires tuning two parameters rather than one single parameter as in yeast. In the simulations used for intermediate genes, we decreased the height of the barrier potential from 25 to 2kBT and reduced the rate of spacer enzymes from 0.16 to 0.12 s−1. Other simulation setups were kept the same as for active genes. (PDF) [file pcbi.1008556.s009.pdf]

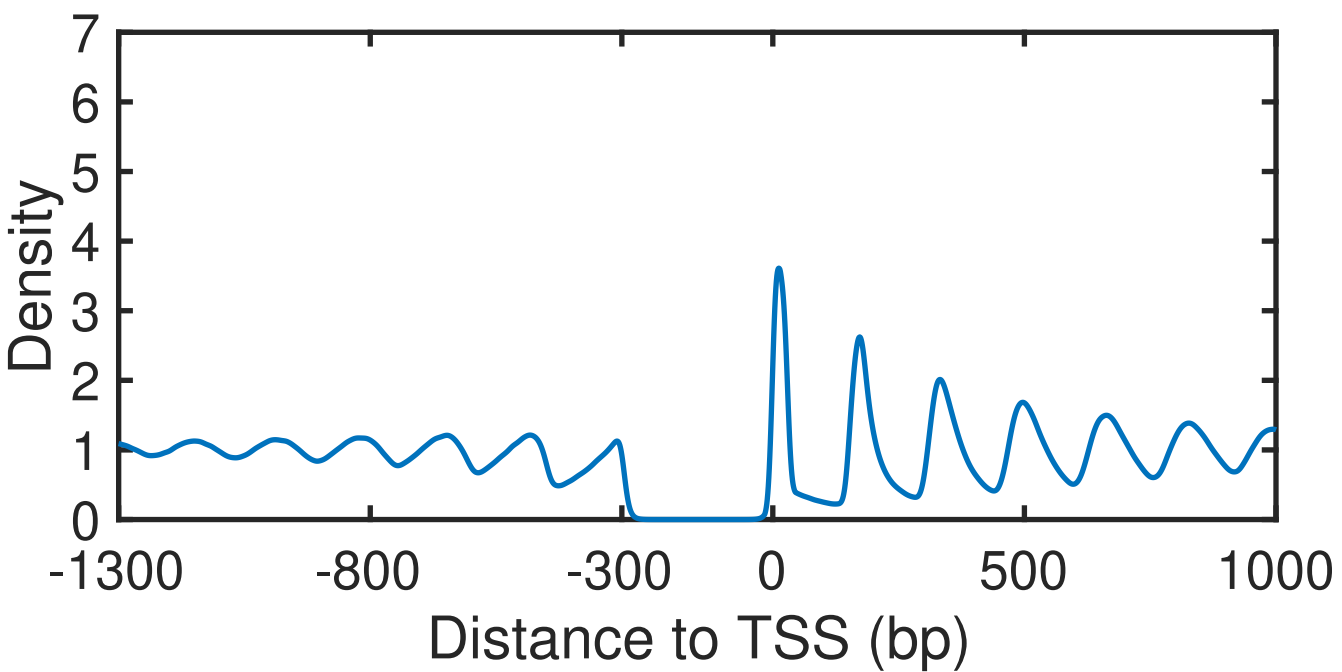

Supplement: S7 Fig — The same data for the blue curve in Fig 5A of the main text was used here to determine the density profile. (PDF) [file pcbi.1008556.s010.pdf]

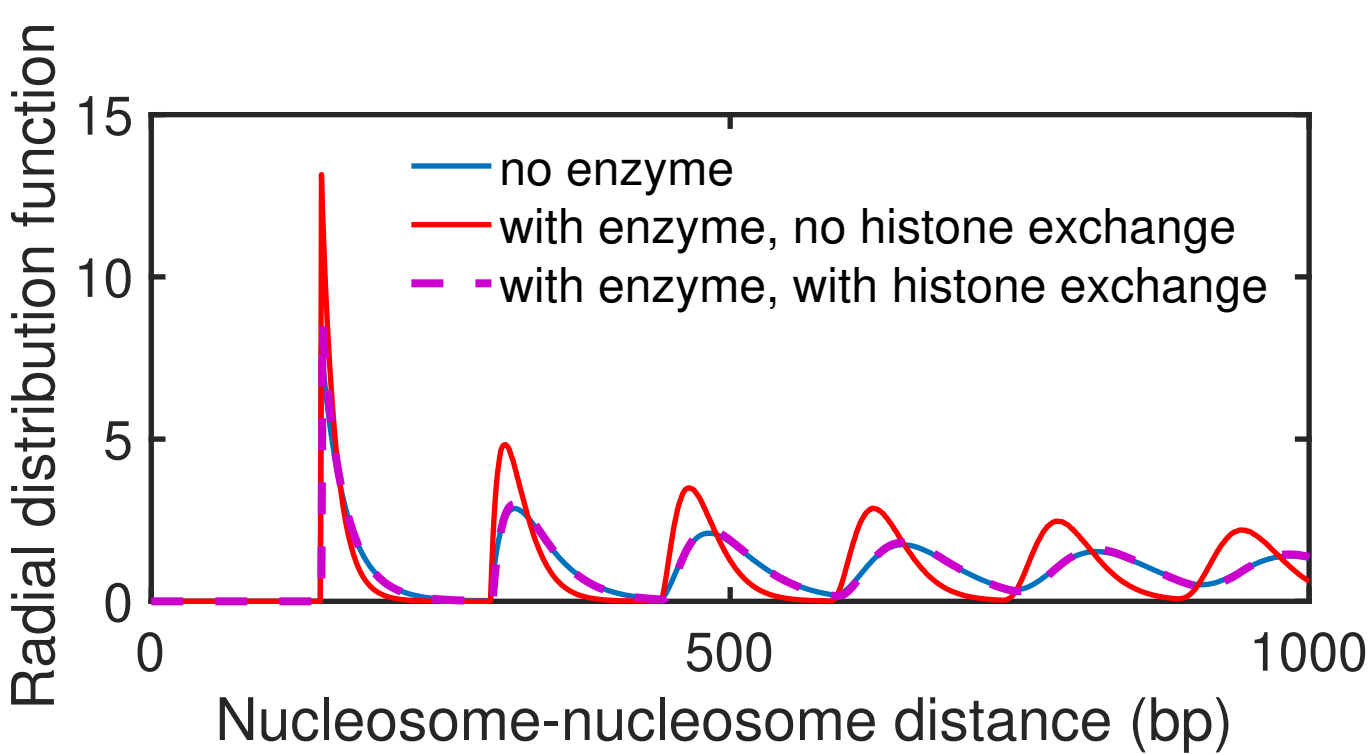

Supplement: S8 Fig — To measure the inter-nucleosome spacing, we determined the radial distribution profiles from the same data used to compute the nucleosome density profiles shown in Fig 6A of the main text. The result from a kinetic model with spacer enzymes and histone exchange (purple) is almost identical to that from a model with no enzymes (blue) and does not exhibit any decrease in inter-nucleosome spacing. (PDF) [file pcbi.1008556.s011.pdf]

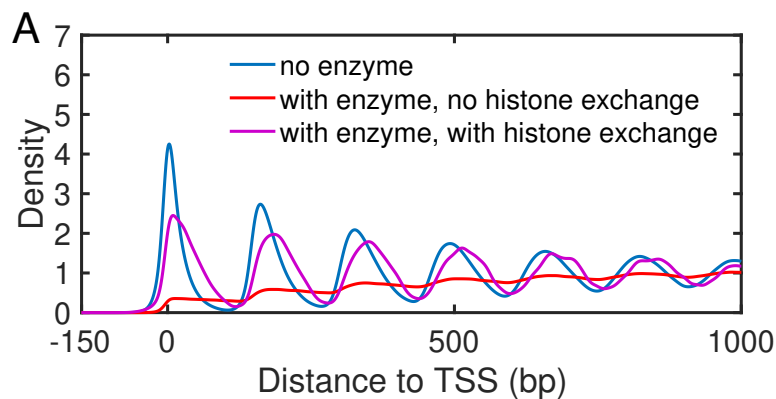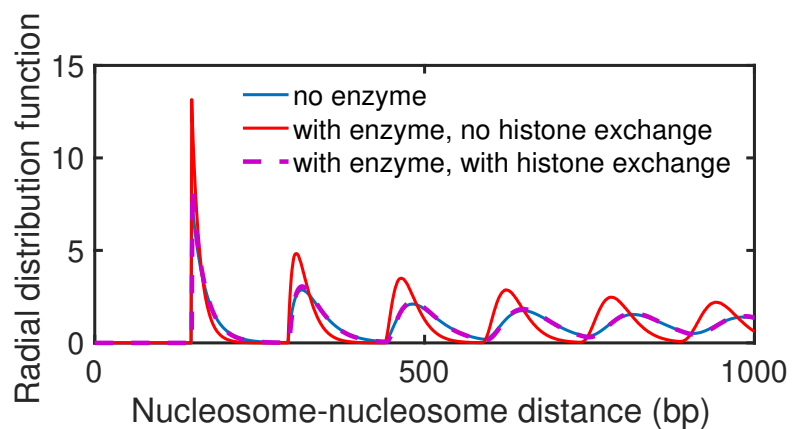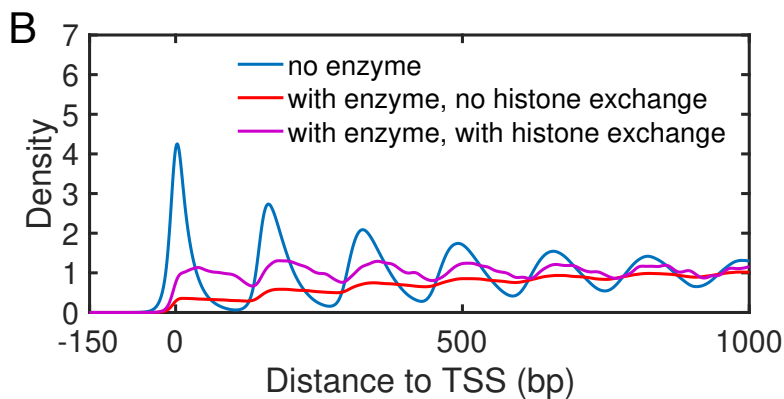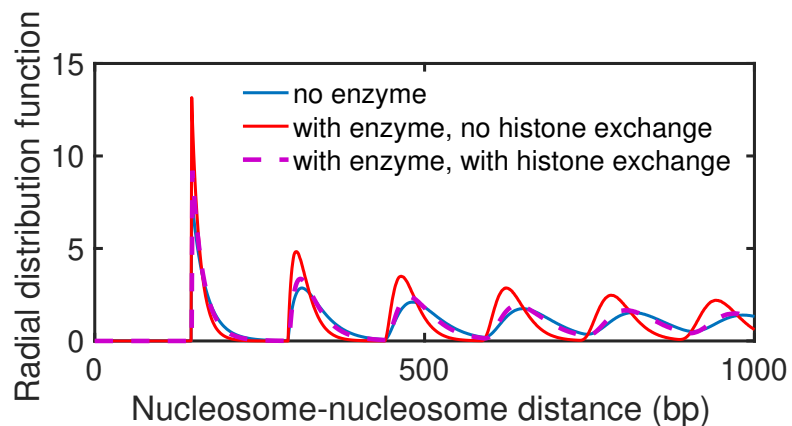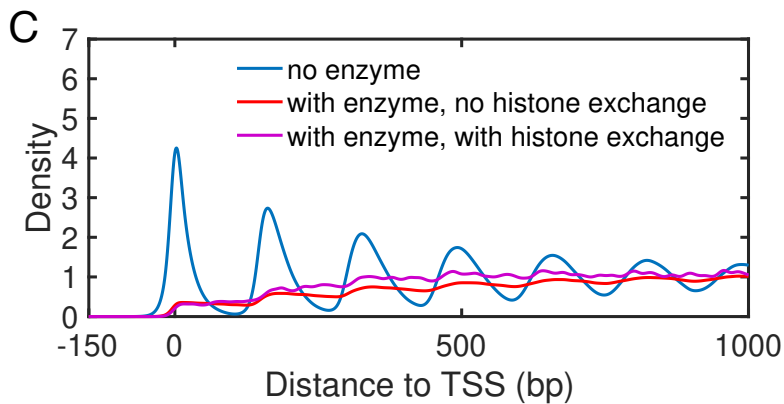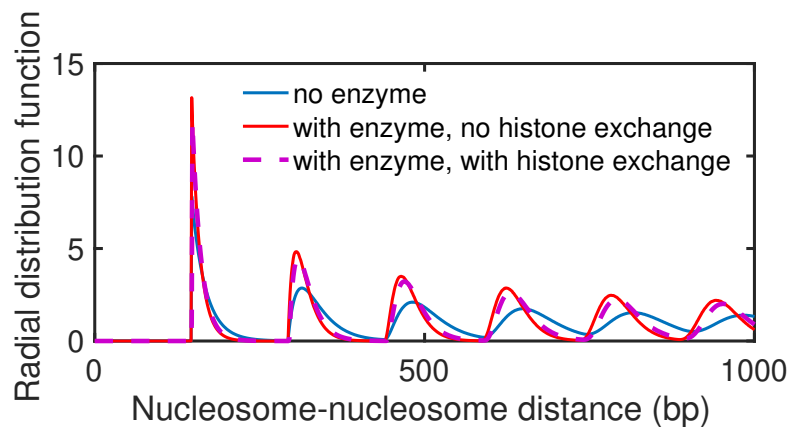

Supplement: S9 Fig — (A) 10−2 s−1. (B) 10−6 s−1. (C) 10−8 s−1. The radial distribution profiles for the kinetic models without enzyme and with enzyme but no histone exchange are identical to those shown in S8 Fig. (PDF) [file pcbi.1008556.s012.pdf]

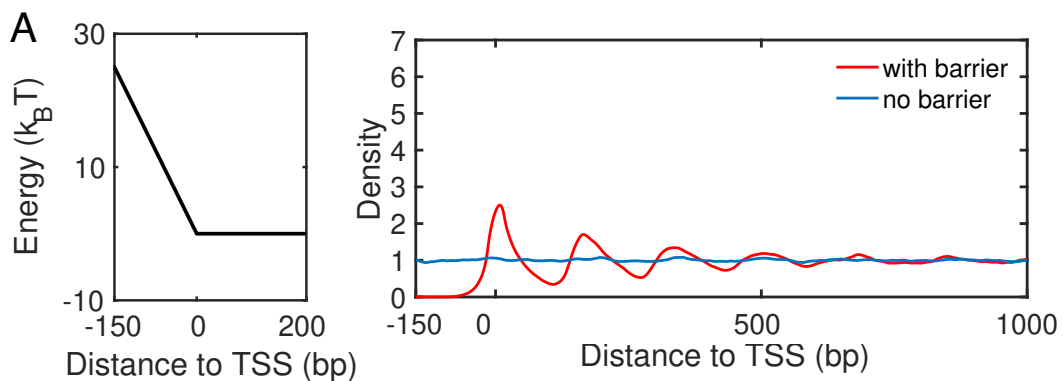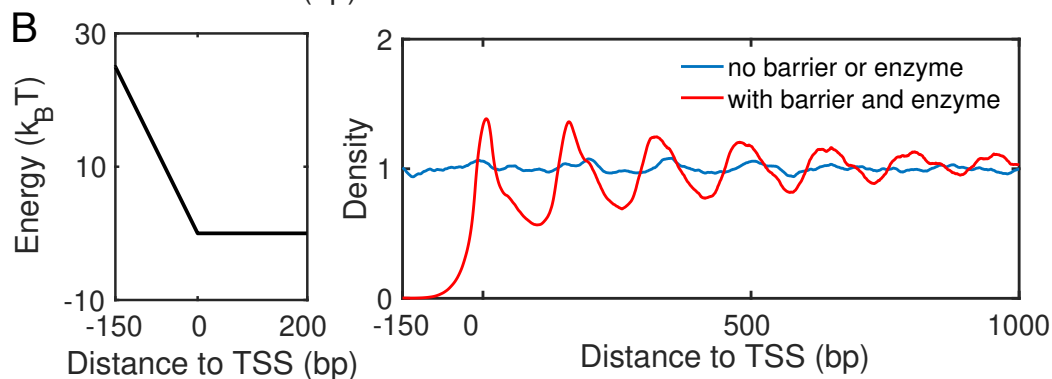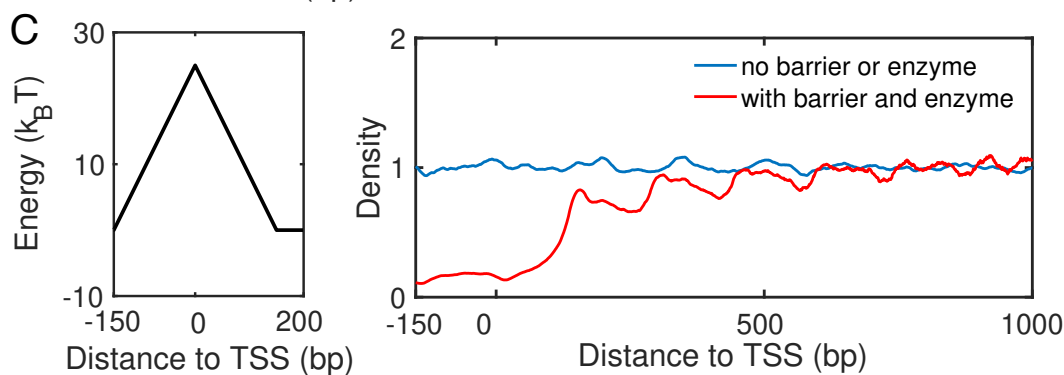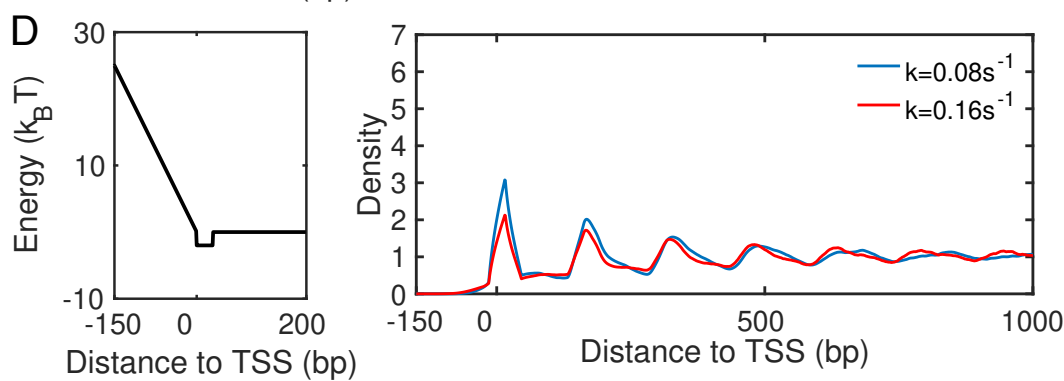

Supplement: S10 Fig — (A) The corresponding plot to Fig 3C of the main text. The blue line was computed without the barrier potential and is identical to the mouse result shown in Fig 3B of the main text. We used the nucleosome binding profiles of mouse genes in the corresponding simulations. The red line was obtained from simulations with the barrier potential and the nucleosome binding profiles of S. cerevisiae genes. (B,C) The corresponding plots to Fig 4A of the main text. Simulations performed for part B used the same set up as those in the main text, except with the addition of nucleosome binding profiles computed using mouse genes. Simulations performed for part C uses a shifted promoter potential and a nucleosome density of 0.78. Both of these two changes are supported by the experimental data shown in Fig 1 of the main text. (D) The corresponding plot to Fig 5A of the main text. Simulations were performed using the same set up as those in the main text, except with the addition of nucleosome binding profiles computed using yeast genes. See text Simulations with DNA sequence specific nucleosome binding in S1 Supporting information for simulation details. (PDF) [file pcbi.1008556.s013.pdf]

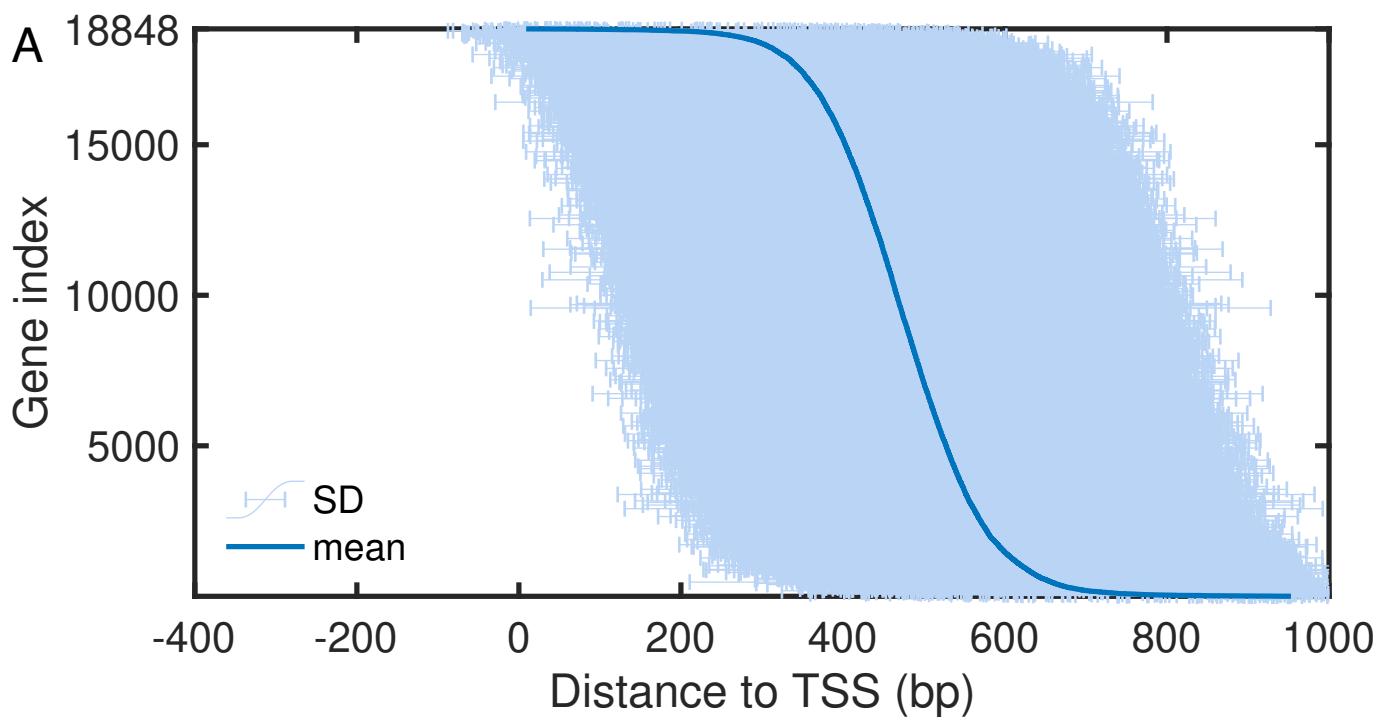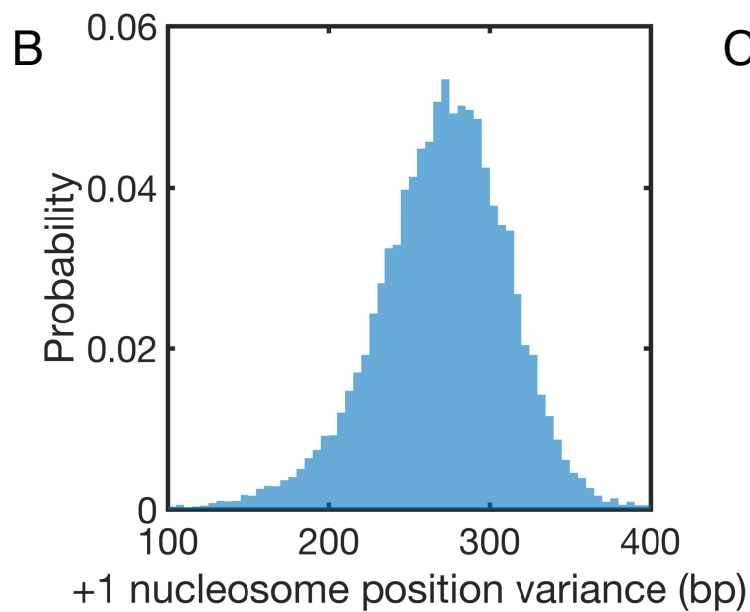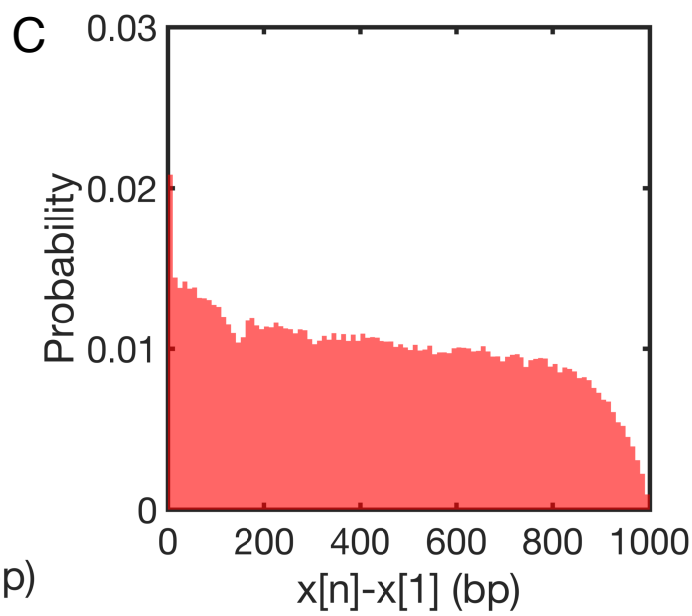

Supplement: S11 Fig — The single-cell nucleosome mapping data for mouse ESC was obtained from the NCBI database with accession number GSE96688 [39]. The +1 nucleosome was defined as the first nucleosome in the downstream 1000 bp region of TSS. Only mapped nucleosomal fragments with a length of 140-180 bp were retained, and the centers of fragments were considered as the nucleosome dyad position. We further discarded segments for which the first nucleosome is more than 1000 bp away from the TSS. This treatment left out 121 genes (18,969 in total) for which no nucleosomes were found in all samples within 1000 bp of TSS. (A) Genes exhibit large variance in the position of the +1 nucleosomes. Each row represents an individual gene, and they were sorted based on the mean distance between +1 nucleosome and TSS. The solid line correspond to the mean position and the light blue errorbars represent standard deviations. (B) Probability distribution of the variance shown in part A. (C) Probability distribution of distance between +1 nucleosome position of a given gene x[n] and the smallest position of that gene found across all cells x[1]. We note that a potential caveat of single cell experiments is that they might suffer from sparse data coverage. Failing to detect some nucleosomes could misguide data analysis, causing the assignment of +2 or +3 as +1 nucleosome and an exaggeration of the variance seen in part A. Though it would be difficult to rule out artifacts from missing data, we can gauge their impact by considering extreme cases. In particular, we consider the case that all nucleosome arrays in different cells have identical +1 nucleosome positions, and the variance arises purely from missing data and misassignment. In this case, the probability distribution of x[n] − x[1] should exhibits clear peaks separated by nucleosome repeat length. As shown in part C, no significant peaks can be seen in the distribution. Therefore, the +1 nucleosome position variance should not arise purely as a res [file pcbi.1008556.s014.pdf]

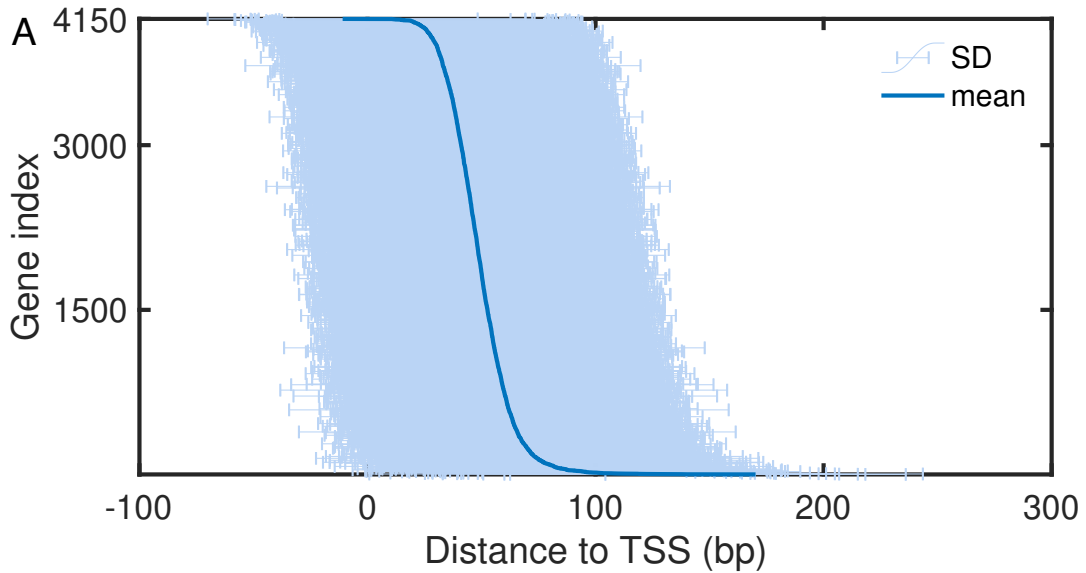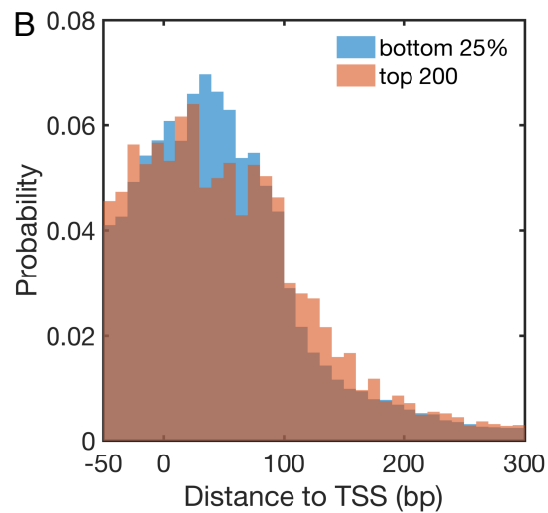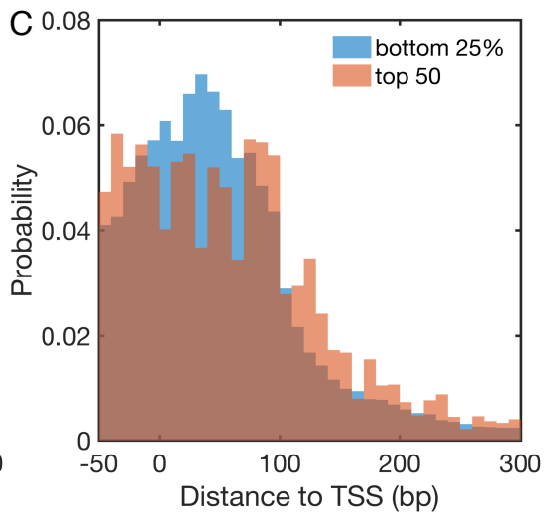

Supplement: S12 Fig — Data produced from the Au group [45] were used for the analysis. The +1 nucleosome was defined as the first nucleosome found in the [-50, 1000] bp region around TSS. We included 50 bp upstream of the TSS since the +1 nucleosomes peak in yeast is closer to the TSS than mouse ESC, as is evident in Fig 1 of the main text. (A) Variance of +1 nucleosome position across different genes. Each row represents an individual gene, and they were sorted based on the mean distance between +1 nucleosome and TSS. The solid line correspond to the mean position and the light blue errorbars represent standard deviations. (B,C) Probability distribution of the +1 nucleosome position for various group of genes. (PDF) [file pcbi.1008556.s015.pdf]

**A**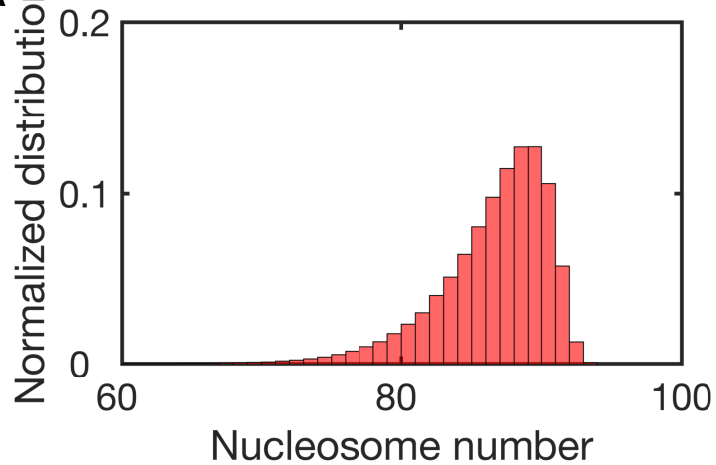**B**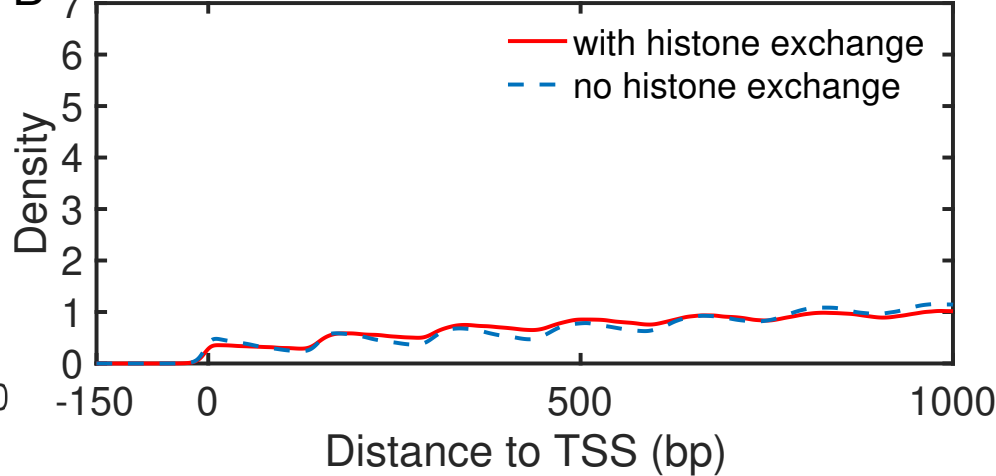

Supplement: S13 Fig — (A) Probability distribution for the number of nucleosomes bound to the DNA computed from the simulation data used for creating Fig 4A (red) of the main text. Over 90% of the simulated configurations falls into the ±10 interval of the peak nucleosome number 86. We note that the length of free DNA region is only 98 nucleosomes due to the presence of the barrier potential in the promoter region. The peak of the distribution with 86 nucleosomes, therefore, provides a density of approximately 0.88. (B) Comparison between nucleosome density profiles obtained from artificial kinetics (red) and from rigorous stochastic simulations of diffusion and enzyme remodeling (blue). In these additional simulations, no explicit histone exchange was considered, and the 86 nucleosomes remain bound to the one-dimensional lattice at all time. We performed 500 simulations that lasted for 7.5 × 106 seconds and were initialized from randomly distributed nucleosomes. 1000 configurations were extracted from the last 5 × 105 s of each trajectory at every 500 seconds to compute the density profile. The good agreement between the two density profiles provide strong support for the accuracy of the algorithm with artificial kinetics used in the main text. (PDF) [file pcbi.1008556.s016.pdf]
